# Toll pathway modulates TNF-induced JNK-dependent cell death in *Drosophila*

**DOI:** 10.1098/rsob.140171

**Published:** 2015-07-22

**Authors:** Chenxi Wu, Changyan Chen, Jianli Dai, Fan Zhang, Yujun Chen, Wenzhe Li, José Carlos Pastor-Pareja, Lei Xue

**Affiliations:** 1 Institute of Intervention Vessel, Shanghai 10th People's Hospital, Shanghai Key Laboratory of Signaling and Disease Research, School of Life Science and Technology, Tongji University, 1239 Siping Road, Shanghai 200092, People's Republic of China; 2 School of Life Science and Technology, Tongji University, 1239 Siping Road, Shanghai 200092, People's Republic of China; 3 School of Life Sciences, Tsinghua University, Medical Science Building, D224, Beijing 100084, People's Republic of China

**Keywords:** cell death, *Drosophila*, Eiger, c-Jun N-terminal kinase, Toll

## Abstract

Signalling networks that control the life or death of a cell are of central interest in modern biology. While the defined roles of the c-Jun N-terminal kinase (JNK) pathway in regulating cell death have been well-established, additional factors that modulate JNK-mediated cell death have yet to be fully elucidated. To identify novel regulators of JNK-dependent cell death, we performed a dominant-modifier screen in *Drosophila* and found that the Toll pathway participates in JNK-mediated cell death. Loss of Toll signalling suppresses ectopically and physiologically activated JNK signalling-induced cell death. Our epistasis analysis suggests that the Toll pathway acts as a downstream modulator for JNK-dependent cell death. In addition, gain of JNK signalling results in Toll pathway activation, revealed by stimulated transcription of *Drosomycin* (*Drs*) and increased cytoplasm-to-nucleus translocation of Dorsal. Furthermore, the Spätzle (Spz) family ligands for the Toll receptor are transcriptionally upregulated by activated JNK signalling in a non-cell-autonomous manner, providing a molecular mechanism for JNK-induced Toll pathway activation. Finally, gain of Toll signalling exacerbates JNK-mediated cell death and promotes cell death independent of caspases. Thus, we have identified another important function for the evolutionarily conserved Toll pathway, in addition to its well-studied roles in embryonic dorso-ventral patterning and innate immunity.

## 1. Background

The excellent work for studying the pivotal functions of the Toll/IL-1R pathway in innate immunity in *Drosophila* and mammals earned Jules A. Hoffmann and Bruce A. Beutler the Nobel Prize in Medicine in 2011. Toll was initially identified in *Drosophila* as a type Ι trans-membrane receptor required for establishing the dorsal–ventral polarity during embryonic development [1,2]. Other components of the Toll pathway, including Spätzle, Tube, Pelle, Cactus and Dorsal, were also characterized as crucial regulators of dorsal–ventral patterning [3–9]. Subsequently, the Toll signalling pathway was implicated in host resistance against fungal and Gram-positive bacterial infections [10–12], which triggers in the fat body the production of antimicrobial peptides (AMPs), among which the antifungal peptide *Drosomycin* (*Drs*) appears to be the principal target of the Toll pathway [10,13]. To activate the Toll pathway in development or immunity, the first step is to cleave the inactive precursor of the Toll receptor ligand Spätzle (Spz) [8,9]. Upon binding to the active Spz ligand, Toll receptor recruits Tube and the kinase Pelle through the adaptor protein myeloid differentiation primary response protein 88 (MyD88), to assemble a receptor-proximal oligomeric complex [14,15]. Activation of Pelle triggers the phosphorylation and degradation of *Drosophila* IκB factor Cactus, which sequesters Dorsal and Dif (Dorsal-related immunity factor), the NF-κB factors in *Drosophila*, in the cytoplasm [16]. Once Cactus is degraded in response to the signal, Dorsal and Dif translocate to the nucleus and activate the transcription of target genes [17]. The innate immune system, which serves as the first-line defence against pathogen infection, appeared early in evolution and is highly conserved in metazoans [18,19]. To date, there are 10 Toll-like receptors (TLRs) in humans and 12 TLRs in mice, which all activate NF-κB factors in a MyD88-dependent manner to induce a set of immune responses, such as inflammation [20,21]. Although the Toll/TLR pathway has been conserved in evolution, mammalian TLR signalling is not involved in development, whereas the *Drosophila* Toll pathway plays pivotal roles in both development and immunity [10,22,23].

The c-Jun N-terminal kinase (JNK) represents one sub-group of the MAP kinase (MAPK) family [24,25]. The JNK pathway has been evolutionarily conserved from fly to human, and is involved in the regulation of a wide range of cellular activities including proliferation, differentiation, migration and apoptosis [26,27]. In *Drosophila*, the tumour necrosis factor (TNF) orthologue Eiger (Egr) triggers cell death through its receptor Grindelwald (Grnd) [28] and the TNF receptor-associated factor 2 (dTRAF2), which in turn activates the conserved JNK cascade including the JNKK kinase dTAK1 (MAP3K), the JNK kinase hemipterous (Hep, MAP2K) and Basket (Bsk) that encodes the *Drosophila* JNK (MAPK) [29–35]. The activated JNK phosphorylates and activates transcription factors including the AP-1 family members Jun and Fos, which are encoded by the *jra* and *kayak* genes in *Drosophila,* respectively [36–39]. JNK signalling also activates another transcription factor forkhead box O (FoxO), which promotes cell death by upregulating the transcription of the pro-apoptotic gene *head involution defective* (*hid*) [36–39]. Although recent studies have identified additional components in this pathway [40–44], the regulating network centred on JNK in modulating cell death, as well as the underlying mechanisms, have not been fully elucidated.

Associations among the TNF/JNK pathway, immune signalling and cell death have been reported in *Drosophila* [45–48], yet the conclusions are controversial, and the underlying mechanisms remain elusive. Seong *et al*. [46] found low-dose radiation (LDR) induces both Toll signalling-mediated innate immunity and activation of the JNK pathway, yet a potential interaction between the Toll and JNK pathways was not investigated. In contrast to the Toll signalling that is activated by fungi and Gram-positive bacteria, the Immune Deficiency (Imd) pathway is predominantly activated by Gram-negative bacteria [49,50]. Bangi *et al*. [45] suggested that an Imd–dTab2-dTAK1-JNK signalling is involved in bacterial-induced invasion and dissemination of oncogenic hindgut cells. However, the Vidal laboratory reported that tumours trigger a systemic immune response through the Egr pathway, which upregulates Toll signalling in the fat body. This activation of Toll, in turn, is required to induce tumour cell death through haemocyte-derived Egr, whereas the Imd pathway is not implicated in this crosstalk [47]. Together, these studies suggest that the Toll pathway may interact with Egr–JNK signalling in regulating cell death during tumour development, yet the modulation mechanisms and a direct role of Toll signalling in cell death have not been documented.

In this study, we have characterized the Toll pathway as an essential modulator of Egr-induced JNK-mediated cell death in *Drosophila*. First, loss of Toll signalling blocks Egr-induced JNK-mediated cell death in eyes and wings. Second, the Toll pathway acts downstream of FoxO to modulate Egr-triggered JNK-mediated cell death. Third, gain of JNK signalling induces Toll pathway activation, indicated by nuclear accumulation of Dorsal and transcriptional activation of *Drosomycin*. Furthermore, JNK signalling activates the Toll pathway through transcriptional upregulation of the Spz family ligands in a non-cell-autonomous manner. Finally, gain of Toll signalling promotes cell death and synergistically enhances Egr-triggered cell death. In conclusion, we have identified a previous unknown function of the Toll pathway in modulating TNF-induced JNK-dependent cell death, in addition to its well-established roles in dorsal–ventral patterning and immunity.

## 2. Results

### 2.1. Depletion of Toll signalling suppresses Egr-induced cell death in eye development

**Figure 1.**
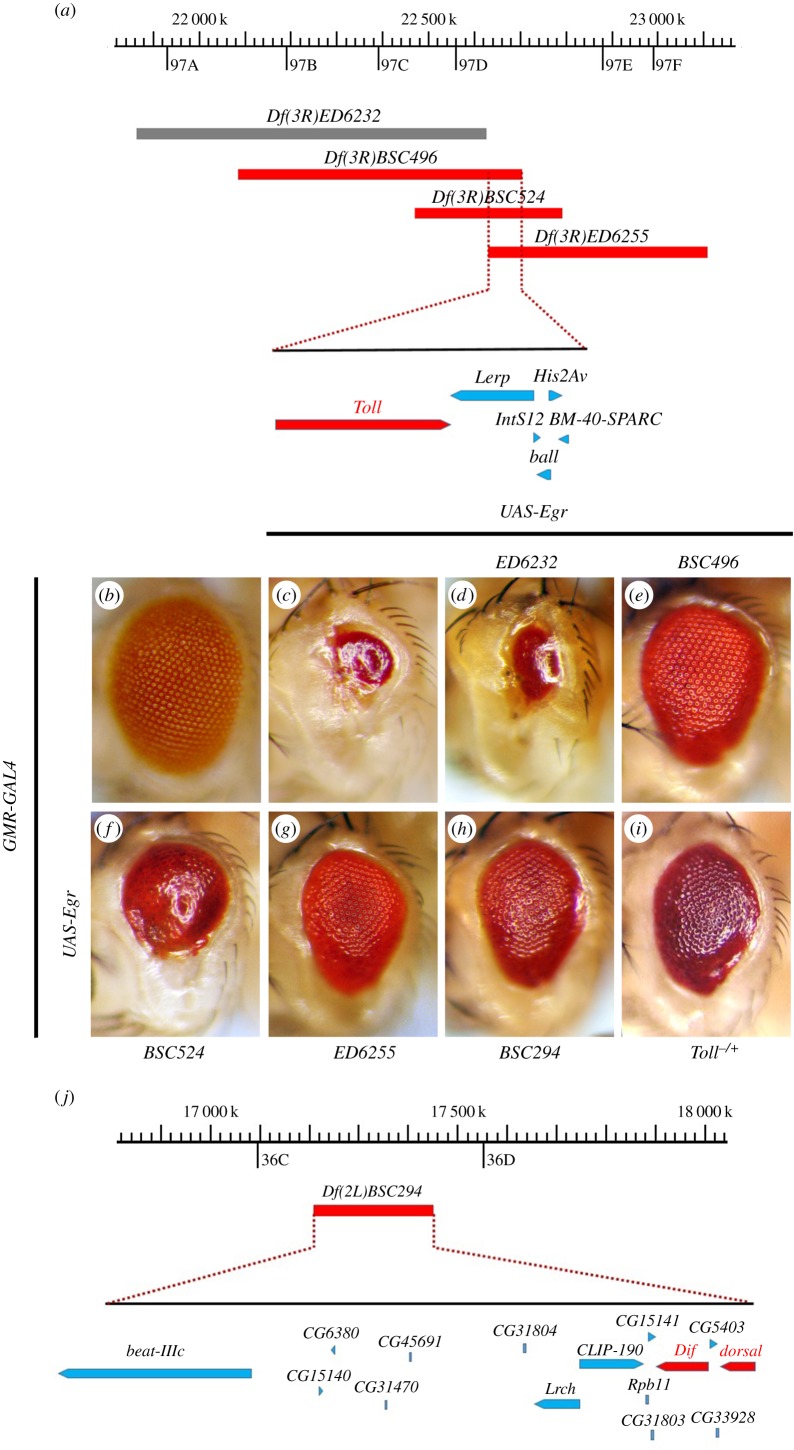
A genetic screen for dominant modifiers of *GMR*>*Egr*-induced eye-ablation phenotype. (*a* and *j*) Schematic of the genomic region surrounding *Toll* or *Dif* and *dorsal* locus. The deleted regions uncovered by deficiencies are indicated in grey (no effect) or red (suppressor). (*b–i*) Light micrographs of *Drosophila* adult eyes are shown. Compared with *GMR*-*GAL4* control (*b*), *GMR*>*Egr-*triggered small eye phenotype (*c*) remains unaffected by *Df(3R)ED6232* (*d*), but is partially inhibited by deficiencies *Df(3R)BSC496* (*e*), *Df(3R)BSC524* (*f*), *Df(3R)ED6255* (*g*), *Df(3R)BSC294* (*h*) or *Toll^r3^*mutation (*i*). See the electronic supplementary material for detailed genotypes.

**Figure 2.**
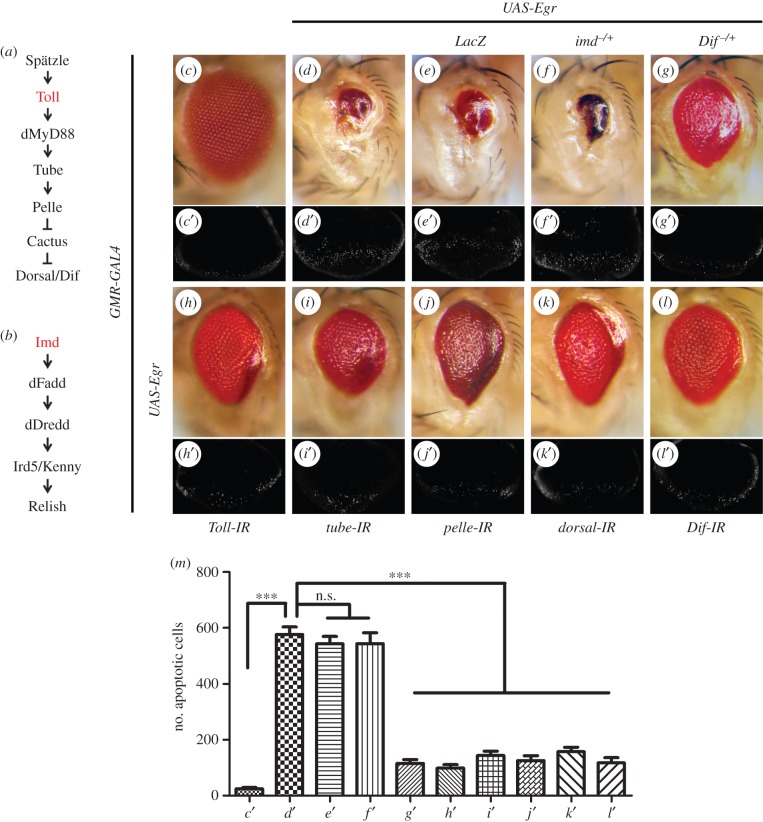
Loss of Toll signalling inhibits *GMR*>*Egr*-triggered cell death. (*a*,*b*) Diagrams for the key components of Toll and Imd signalling. Light micrographs of *Drosophila* adult eyes (*c–l*) and fluorescence micrographs of third-instar larval eye discs (*c*′–*l*′) are shown. Compared with control (*c* and *c*′), *GMR*>*Egr*-induced small eye phenotype (*d*) and cell death in eye discs (*d*′) remain unaffected by expression of LacZ (*e* and *e*′) or mutation in *imd* (*f* and *f*′), but are partially suppressed by mutation in *Dif* (*g* and *g*′) or RNAi-mediated downregulation of Toll pathway components: *Toll*, *tube*, *pelle*, *dorsal* and *Dif* (*h–l* and *h*′–*l*′). (*m*) Statistic analysis of cell death in eye discs shown in (*c*′–*l*′). Average number of dying cells labelled by AO staining are counted. Error bars indicates standard deviation. One-way ANOVA with Bonferroni multiple comparison test was used to compute *p*-values, significance was indicated with asterisks (***p < 0.001, *n* = 10 in each group); n.s., not significant. See the electronic supplementary material for detailed genotypes.

Ectopic expression of Egr in *Drosophila* eyes driven by *GMR*-*GAL4* leads to vastly reduced adult eye size (figure 1*b,c* and 2*c,d*) [29,30], resulting from extensive cell death posterior to the morphogenetic furrow (MF) in third-instar eye discs (figure 2*c*′,*d*′), visualized by acridine orange (AO) staining that detects dying cells [51]. To identify additional regulators of Egr-triggered cell death, we performed a genetic screen for dominant modifiers of the *GMR*>*Egr* small-eye phenotype using the Bloomington *Drosophila* Stock Center Deficiency kit that covers more than 95% of the genome. Once a deficiency was found to enhance or suppress the *GMR*>*Egr* eye-ablation phenotype, additional overlapping deficiencies were used for fine-mapping of the candidate gene [40,42,52]. One of the suppressors was mapped cytologically within 97D2–97D4, a region uncovered by three overlapping deficiencies: *Df(3R)BSC496*, *Df(3R)BSC524* and *Df(3R)ED6255* (figure 1*a*). The *GMR*>*Egr* eye phenotype is partially suppressed by the three deficiencies (figure 1*e–g*), but not by the adjacent *Df(3R)ED6232* (figure 1*d*). The region of 97D2–97D4 contains six genes including *Toll* (figure 1*a*), the *Drosophila* homologue of TLRs, that encodes a type Ι trans-membrane receptor involved in the activation of NF-κB in dorsal–ventral patterning and immunity [3,10,21]. Accordingly, the *GMR*>*Egr* eye phenotype is suppressed to a similar extent by deleting half of the dosage of *Toll* (figure 1*i*) or expressing a *Toll* RNAi (figure 2*h*). In addition, loss of *Toll* significantly suppressed *GMR*>*Egr-*triggered cell death in eye imaginal discs (figure 2*h*′,*m*). Thus, the Toll receptor is required for Egr-triggered cell death in eye development.

From the same screen, we also identified *Df(2L)BSC294* as a suppressor of *GMR*>*Egr-*induced eye phenotype (figure 1*h*). This small deficiency uncovers 36C2–36C9, a region that harbours 15 genes including *dorsal* and *Dif* (figure 1*j*), both of which encode the *Drosophila* NF-κB factor operating in the Toll pathway (figure 2*a*). Consistently, *GMR*>*Egr-*triggered cell death in eye discs and adult eye phenotype (figure 2*d*,*d*′) are partially inhibited by removing one copy of endogenous *Dif* (figure 2*g*,*g*′,*m*), or RNAi-mediated knocking-down of *dorsal* or *Dif* (figure 2*k*,*k*′,*l*,*l*′,*m*), but not of *LacZ* (figure 2*e*,*e*′,*m*), suggesting the involvement of NF-κB in Egr-triggered cell death.

Next, we extended our curiosity to other components of the Toll/NF-κB pathway (figure 2*a*). We found that both the small eye phenotype and increased cell death induced by *GMR*>*Egr* were notably inhibited by RNAi-mediated knocking-down of *tube* or *pelle* (figure 2*i*,*i*′,*j*,*j*′,*m*). Taken together, these data imply that the Toll pathway plays an essential role in Egr-triggered cell death.

Both Toll and Imd pathways are implicated in *Drosophila* innate immune response against microbial infection. The Toll pathway is activated primarily by Gram-positive bacteria and fungi while the Imd pathway responds mainly to Gram-negative bacteria infection (figure 2*b*) [13,53]. As Egr-triggered cell death depends on JNK signalling [29,30], which is also involved in the immune response of the Imd pathway [54,55], we examined a potential role of the Imd pathway in Egr-induced cell death. We found that *GMR*>*Egr-*induced cell death phenotypes were not suppressed by knocking-down *imd* or *relish* (electronic supplementary material, figure S1*a–c*), or deleting one or two copies of the endogenous *imd* (figure 2*f*,*f*′,*m*; electronic supplementary material, figure S1*d*,*f*), or homozygous mutation *of relish* (electronic supplementary material, figure S1*g*,*i*), as well as expression of LacZ (electronic supplementary material, figure S1*e*,*h*), suggesting the Imd pathway is dispensable for Egr-induced cell death.

### 2.2. Loss of Toll signalling suppresses JNK-mediated cell death in eye development

**Figure 3.**
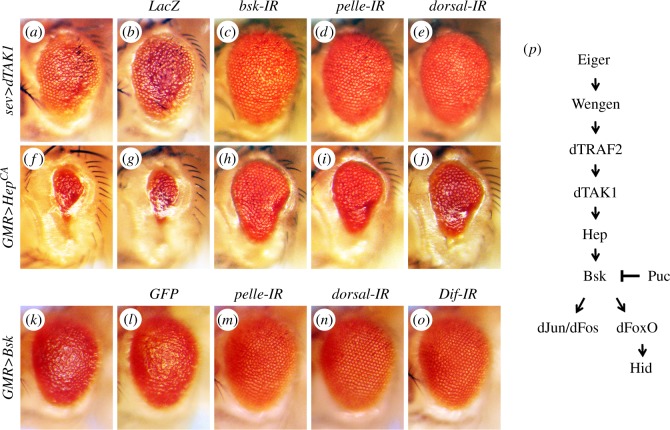
Toll signalling acts downstream of JNK in eye development. (*a–o*) Light micrographs showing *Drosophila* adult eyes. The small and rough eye phenotype resulting from ectopic expression of dTAK1 (*a*) or Hep^CA^ (*f*) is suppressed partially by RNAi-mediated knocking-down of *bsk* (*c* and *h*), *pelle* (*d* and *i*) or *dorsal* (*e* and *j*), but not of *LacZ* (*b* and *g*). The rough eye phenotype produced by *GMR*>Bsk (*k*) is obviously suppressed by RNAi-mediated inactivation of Toll pathway components: *pelle*, *dorsal* and *Dif* (*m–o*), but not by the expression of GFP (*l*). (*p*) A diagram for the key components of the Egr–JNK pathway. See the electronic supplementary material for detailed genotypes.

Egr-triggered cell death is mainly mediated by JNK signalling [29,30]. To genetically map the epistatic relationship between the Toll pathway and JNK cascade (figure 3*p*), we first examined the genetic interplays between the Toll pathway and dTAK1 (JNKKK) or Hep (JNKK) in the eye. As expression of dTAK1 driven by *GMR*-*GAL4* (*GMR*>*dTAK1*) results in pupa lethality [31], probably caused by the leaky expression of *GMR*-*GAL4* in other tissues including brain, wing and leg discs [56] we used *sev*-*GAL4*, another eye specific driver, to express dTAK1 in the eye. Eye specific expression of dTAK1 driven by *sevenless* (*sev*)-*GAL4* or a constitutive activated form of Hep (Hep^CA^) driven by *GMR*-*GAL4* induces JNK-mediated cell death and generates rough eyes with reduced size (figure 3*a,f*) [31,42]. Both phenotypes are considerably suppressed by knocking-down *pelle* or *dorsal* (figure 3*d,e,i,j*). In the meantime, expression of LacZ (figure 3*b,g*) and a *bsk* RNAi (figure 3*c,h*) were included as a negative and positive control, respectively. Moreover, ectopic expression of Bsk (*Drosophila* JNK) under the control of *GMR-GAL4* also produces a small and rough eye phenotype (figure 3*k*), which is clearly suppressed by knockdown Toll signalling components (figure 3*m–o*), but not the expression of GFP (figure 3*l*). Taken together, these data suggest that the Toll pathway acts downstream of JNK in modulating cell death.

### 2.3. Toll signalling modulates JNK-mediated cell death in wing development

**Figure 4.**
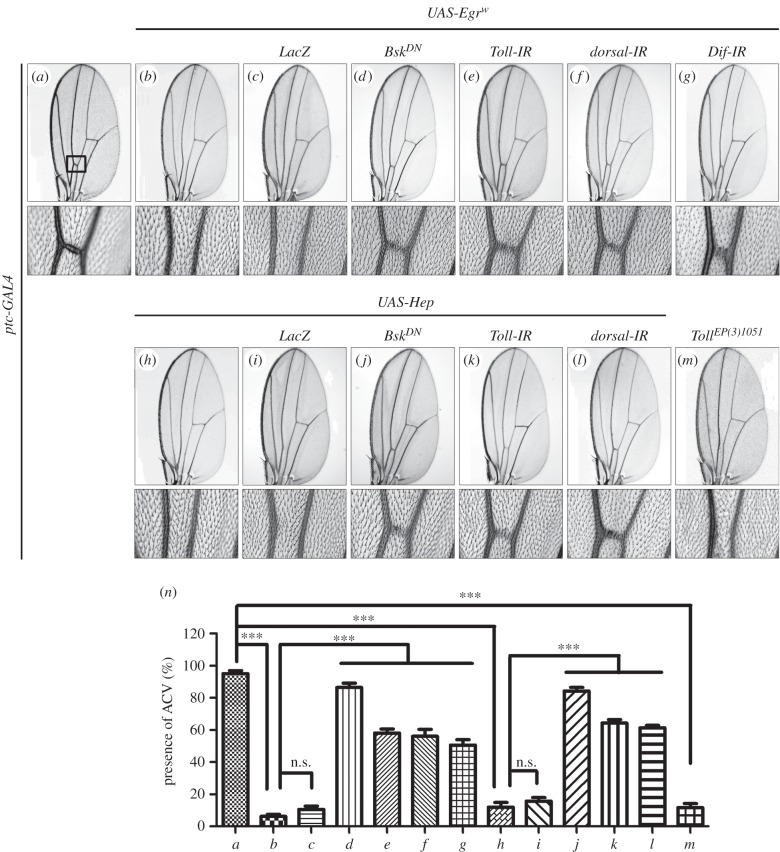
Toll signalling acts downstream of Hep in wing development. Light micrographs of *Drosophila* adult wings (*a*–*m*). Compared with *ptc*-*GAL4* control (*a*), ectopic expression of Egr^w^ (*b*) or Hep (*h*) or Toll (*m*) driven by *ptc*-*GAL4* generates loss of ACV phenotype. This phenotype, produced by *ptc*>*Egr^W^* (*b*) or *ptc*>*Hep* (*h*), is strongly suppressed by a dominant-negative form of Bsk (*d* and *j*) or by depletion of Toll signal (*e–g*, *k* and *l*), but not that of LacZ (*c* and *i*). The lower panels show high magnification view of the boxed area in upper panels (*a–m*). (*n*) Quantification of the ACV phenotype as shown in panels (*a*–*m*) (for each genotype, *n* = 20). Error bars indicates standard deviation. One-way ANOVA with Bonferroni multiple comparison test was used to compute *p*-values, significance was indicated with asterisks (***p < 0.001); n.s., not significant. See the electronic supplementary material for detailed genotypes.

To investigate whether the Toll pathway modulates JNK-mediated cell death in other cellular contexts, we activated JNK signalling in distinct regions of the wing disc. Expression of Egr or Hep along the anterior/posterior (A/P) compartment boundary driven by *ptc*-*GAL4* results in cell death and produces a loss of anterior cross vein (ACV) phenotype (figure 4*a,b,h,n*) [42,57], which mimics the phenotype generated by expressing the cell death gene *grim* (*ptc*>*Grim* + *Tub*-*GAL80^ts^*; electronic supplementary material, figure S2). This phenotype is recapitulated by Toll expression (figure 4*m*), and suppressed by expressing a dominant-negative form of Bsk (Bsk^DN^) (figure 4*d,j*) or RNAi-mediated inactivation of Toll pathway components (figure 4*e–g,k,l*), but not by expressing LacZ (figure 4*c,i*).

Furthermore, expression of Hep in the wing pouch driven by *Scalloped* (*Sd*)-*GAL4* triggers strong cell death (electronic supplementary material, figure S3) that results in severely reduced larval wing disc (electronic supplementary material, figure S4*g,h*) and adult wing blade (electronic supplementary material, figure S4*a,b,f*). Both phenotypes are significantly suppressed by expressing Bsk^DN^ or downregulation of Toll signalling (electronic supplementary material, figure S4*d–f,j,k*), but not by expressing GFP (electronic supplementary material, figure S4*c,f,i*), suggesting the Toll pathway modulates JNK-mediated cell death in a non-tissue-specific manner.

### 2.4. Toll signalling acts downstream of FoxO to modulates JNK-mediated cell death

**Figure 5.**
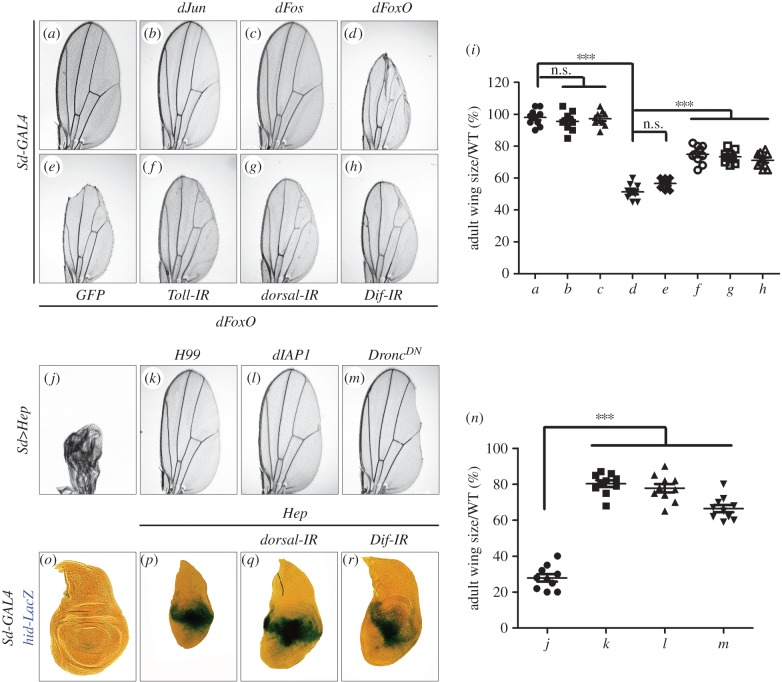
Toll signalling acts downstream of FoxO and mediates caspases-independent cell death. (*a–h* and *j–m*) Light micrographs of *Drosophila* adult wings are shown. Compared with *Sd*-*GAL4* control (*a*), ectopic expression of dFoxO results in an evident reduction in wing size (*d*), which is suppressed significantly by loss of Toll signalling (*f–h*), but not that of GFP (*e*). Expression of dJun or dFos driven by *Sd*-*GAL4* does not produce any visible defects (*b* and *c*). The small wing phenotype induced by *Sd*>*Hep* (*j*) is strongly suppressed by *Df(3L)H99* that deletes one copy of the apoptotic genes *reaper*, *hid* and *grim* (*k*), the expression of DIAP1 (*l*) or Dronc^DN^ (*m*). (*i* and *n*) Quantifications of adult wing size/wild-type (WT) ratio shown in figures *a–h* and *j–m*, respectively (*n* = 10). One-way ANOVA with Bonferroni multiple comparison test was used to compute *p*-values, significance was indicated with asterisks (***p < 0.001); n.s., not significant. (*o–r*) X-Gal staining of a *hid*-*LacZ* reporter in wing discs. Compared with the control (*o*), expression of Hep in the wing pouch driven by *Sd*-*GAL4* induces *hid* transcription (*p*), which cannot be suppressed by knocking-down *dorsal* or *Dif* (*q* and *r*). See the electronic supplementary material for detailed genotypes.

In *Drosophila*, the AP-1 family members Jun and Fos, and the forkhead box factor FoxO act downstream of JNK as the transcription factors to mediate cell death [36–39]. To further delineate the interaction between JNK and Toll signalling, we ectopically expressed the three transcription factors in the developing wing by *Sd*-*GAL4*. Expression of dFoxO (*Sd*>*dFoxO*) triggered severe cell death and generated a small wing phenotype (figure 5*a*,*d*,*i*) [58], whereas expression of Jun (*Sd*>*dJun*) or Fos (*Sd*>*dFos*) did not produce any discernible defects in adult wings (figure 5*b*,*c*,*i*). Furthermore, *Sd*>*dFoxO*-induced wing defect could be partially suppressed by knocking-down Toll signalling (figure 5*f–i*), but remained unaffected by the expression of GFP (figure 5*e,i*), implying that Toll signalling acts downstream of dFoxO to modulates JNK-mediated cell death in *Drosophila*.

### 2.5. Toll pathway is dispensable for caspases-mediated cell death

Previous studies have suggested that Egr–JNK signalling is able to trigger two types of cell death: the apoptotic cell death (caspases-dependent) and the non-apoptotic cell death (caspases-independent) [29,30,44]. In *Drosophila*, apoptotic cell death is induced by upregulation of three pro-apoptotic genes, *rpr*, *hid* and *grim*, and is mediated by activation of caspases [59]. Consistent with previous study [60], we found that activation of TNF–JNK signalling in the wing pouch by *Sd*>*Hep* upregulates the transcription of *hid*, visualized by X-gal staining of *hid*-*LacZ* reporter (figure 5*o,p*). In addition, *Sd*>*Hep*-induced small wing phenotype is partially rescued by the deficiency *Df(3L)H99* that deletes *rpr*, *hid* and *grim*, or by expressing the inhibitor of apoptosis protein DIAP1 or a dominant-negative form of Dronc (*Drosophila* caspase-9) (figure 5*j–n*) [59,61,62], suggesting TNF–JNK signalling could induce apoptotic cell death in wing development.

To test whether the Toll pathway is involved in JNK-mediated caspases-dependent cell death, we knocked-down Toll signalling and found that loss of the Toll pathway could partially suppress *Sd*>*Hep*-induced size reduction, but not the transcriptional upregulation of *hid* in the wing disc (figure 5*o–r*). Moreover, ectopic expression of Hid in *Drosophila* eyes driven by *GMR*-*GAL4* triggers caspases-mediated cell death and produces small adult eye phenotype (electronic supplementary material, figure S5*a*), which cannot be rescued by depletion of the Toll signalling pathway (electronic supplementary material, figure S5*b* and *c*). Hence, we conclude that the Toll pathway is dispensable for caspases-mediated cell death.

### 2.6. Toll signalling modulates physiological JNK signalling-mediated cell death

**Figure 6.**
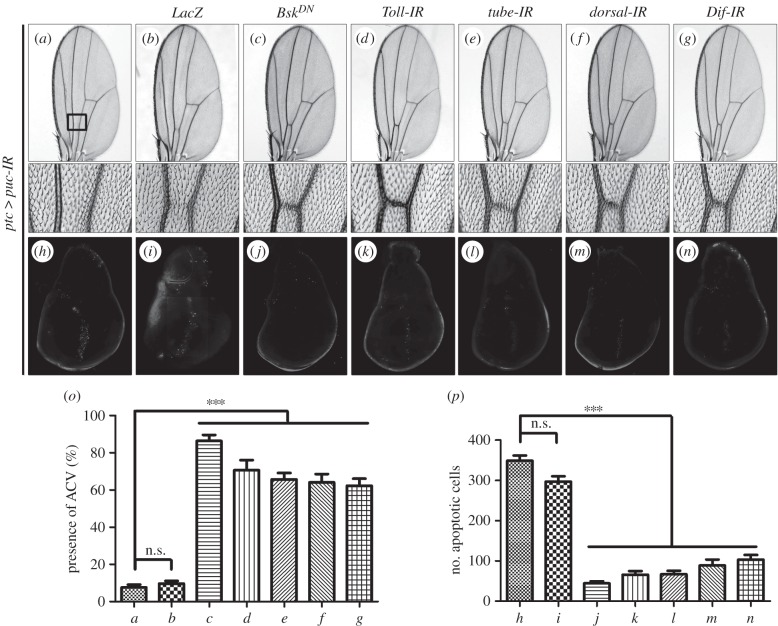
Loss of Toll signalling suppresses physiological JNK-induced cell death. Light micrographs of *Drosophila* adult wings (*a–g*) and fluorescence micrographs of third-instar larval wing discs (*h–n*) are shown. RNAi-mediated downregulation of *puc* along the A/P compartment boundary by *ptc*-*GAL4* produces the loss-of-ACV phenotype in adult wings (*a*), which results from strong cell death in larval wing discs (*h*). Both phenotypes depends on endogenous JNK (*c* and *j*) and the Toll pathway (*d–g* and *k–n*), but not on LacZ (*b* and *i*). The bottom panels show high magnification views of the boxed area in upper panels (*a–g*). (*o* and *p*) Statistical analysis of ACV phenotype (*n* = 20 for each genotype) and cell death in wing discs (*n* = 10) as shown in figures *a–g* and *h–n* respectively. Error bars indicate standard deviation. One-way ANOVA with Bonferroni multiple comparison test was used to compute *p*-values, significance was indicated with asterisks (***p < 0.001); n.s., not significant. See the electronic supplementary material for detailed genotypes.

To investigate whether the Toll pathway is involved in the physiological role of JNK signalling in cell death, we knocked-down *puc*, a negative regulator of JNK activity (figure 3*p*) [63], along the A/P compartment boundary under the control of *ptc*-*GAL4* (*ptc*>*puc-IR*). We observed robust cell death in third-instar larval wing discs and loss of ACV in adult wings (figure 6*a*,*h*,*o*,*p*). Both phenotypes are fully suppressed by expressing the dominant-negative Bsk (figure 6*c*,*j*,*o*,*p*), but remain unaffected by expressing LacZ, which serves as a negative control (figure 6*b*,*i*,*o*,*p*), suggesting loss-of-*puc*-triggered cell death depends on JNK. Furthermore, *ptc*>*puc-IR*-induced cell death and loss-of-ACV phenotypes are suppressed by RNAi-mediated inactivation of Toll pathway components, e.g. *Toll*, *tube*, *dorsal* or *Dif* (figure 6*d–g*, *k–p*), indicating that the Toll pathway is involved in the physiological function of JNK signalling in cell death.

### 2.7. JNK signalling promotes Toll pathway activation

**Figure 7.**
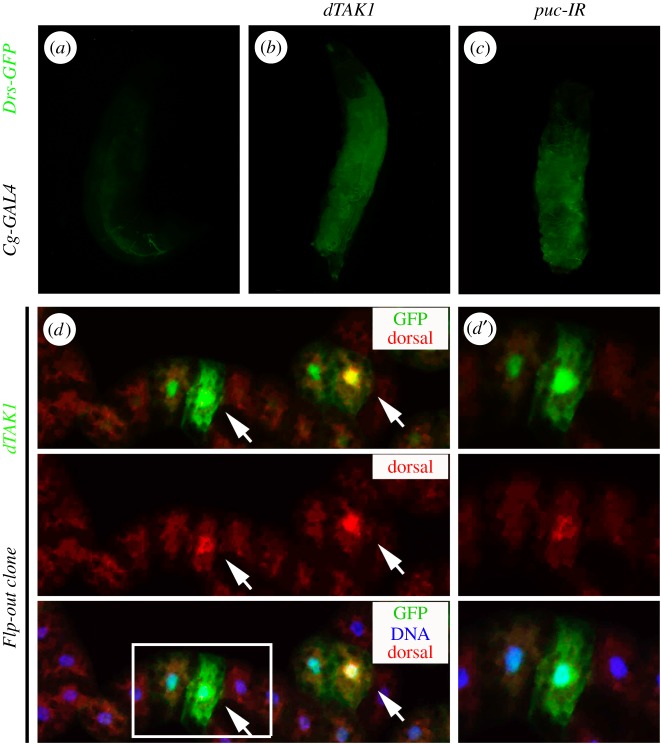
Gain of JNK signalling promotes *Drs* expression and dorsal nuclear localization. (*a–c*) Fluorescent microscope images showing third-instar larvae. Compared with control (*a*), expression of dTAK1 (*b*) or RNAi inactivation of *puc* (*c*) in fat body upregulates *Drs-GFP* expression. (*d*) Fluorescent microscope images showing fat body dissected from third-instar larvae stained with anti-dorsal (red). dTAK1-expressing clones were tagged by GFP (green) and induced for 1 h by heat shock at 37°C and recovered for 24 h at 25°C. Nuclei were labelled with DAPI (blue). Endogenous Dorsal protein displays nuclear localization in cells expressing high level of dTAK1 (arrows). (*d*′) shows high magnification views of the boxed area in (*d*). DAPI, 4, 6-diamidino-2-phenylindole. See the electronic supplementary material for detailed genotypes.

It is known that a fungal infection could trigger the activation of the Toll pathway, which leads to the induction of antifungal peptide *Drs* in the fat body as its principal target [10,13]. To determine whether JNK signalling is sufficient to elicit Toll pathway activation, we examined the expression of *Drs* by a previously described *Drs*-*GFP* reporter [64], and found that expression of dTAK1 driven by the fat body specific *Cg*-*GAL4* resulted in elevated expression of *Drs*-*GFP* (figure 7*a*,*b*). Furthermore, knocking-down *puc* in the fat body also triggers *Drs*-*GFP* expression (figure 7*c*). Hence, both ectopic and physiological JNK activation could induce the transcriptional upregulation of *Drs*, a primary target gene of the Toll pathway.

Our previous data indicated that the Imd pathway is not implicated in TNF/JNK- mediated cell death (figure 2*f*,*f*′,*m* and electronic supplementary material, figure S1). Consistent with these data, activation of JNK signalling did not induce the expression of *Diptercicin* (*Dipt*) (electronic supplementary material, figure S6), a reporter of Imd pathway activity [65–68].

In the non-signalling condition, the I-κB orthologue Cactus retains the NF-κB factor Dorsal in the cytoplasm, inhibiting its nuclear localization and transcription factor activity. Upon activation of Toll signalling, Dorsal is released from Cactus and translocates to the nucleus [69]. To monitor the Toll pathway activity directly, we examined the expression level and subcellular localization of Dorsal *in vivo* by anti-Dorsal staining. Activation of JNK signalling by dTAK1 expression in third-instar eye discs driven by *GMR*-*GAL4* resulted in elevated Dorsal expression posterior to the MF (electronic supplementary material, figure S7). As eye disc cells are too small to distinguish the subcellular distribution of Dorsal protein, we induced ectopic dTAK1-expressing clones in the fat body and examined the nuclear–cytoplasmic shuttling of endogenous Dorsal protein. While Dorsal is mainly localized to the cytoplasm in control cells, it is accumulated in the nuclei and periphery in cells expressing high levels of dTAK1 (figure 7*d*,*d*′). Consistently, we observed increased nuclear accumulation of Dorsal in all fat body cells when JNK signalling was activated by *Cg*-*GAL4*-driven expression of Egr or *puc* RNAi (electronic supplementary material, figure S8). These results imply that gain of JNK signalling is sufficient to trigger the activation of the Toll pathway.

Intriguingly, we found that gain of JNK signalling-triggered Toll pathway activation, visualized by stimulated *Drs-GFP* expression and increased Dorsal nuclear localization, could not be suppressed by expressing caspases inhibitor P35 (electronic supplementary material, figure S9*a–c*), implying that activation of Toll signalling by activated JNK is caspases independent.

### 2.8. Gain of JNK signalling upregulates the transcription of Spz family ligands

**Figure 8.**
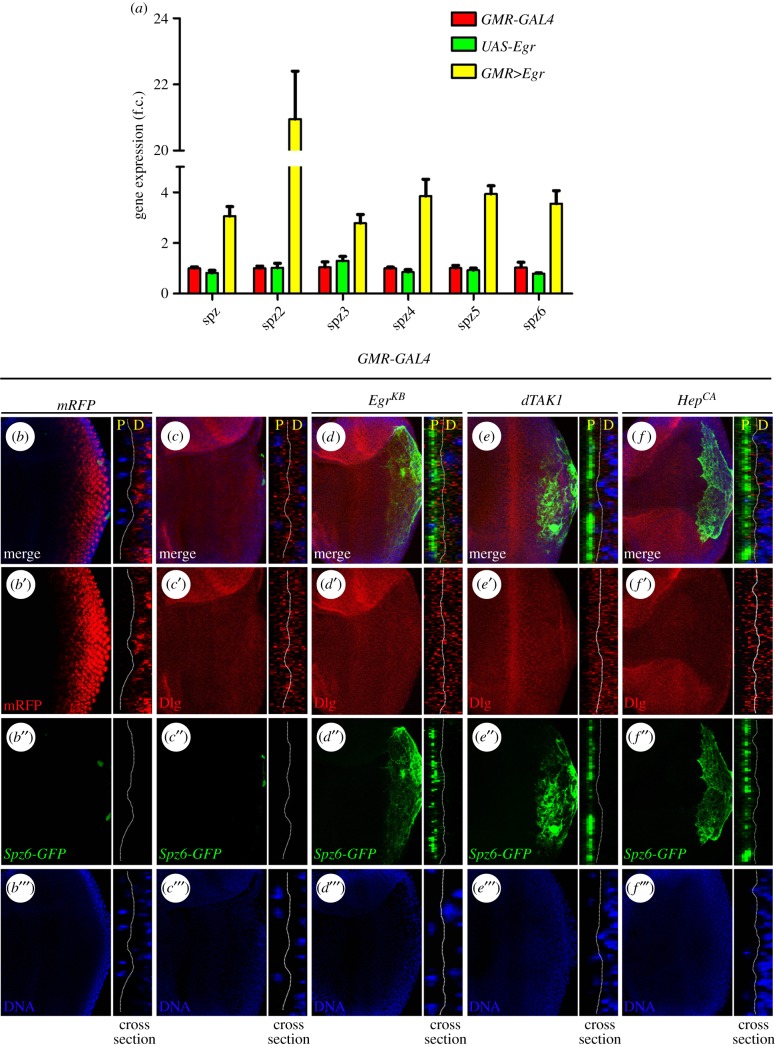
Gain of JNK signalling upregulates the expression of Spz family ligands. (*a*) Histogram showing the levels of *Spz1–6* mRNAs measured by quantitative RT-PCR. Total RNA of *Drosophila* adult eyes was extracted and normalized for cDNA synthesis. Error bars represent standard deviation from three independent experiments. (*b–f*) Fluorescence micrographs of *Drosophila* third-instar larval eye discs are shown. Compared with the *GMR*-*GAL4* control (*c*), *Spz6*-*GFP* expression was evidently increased by expression of Egr (*d*), dTAK1 (*e*) or Hep (*f*), mRFP marking the *GMR*-*GAL4* expression region (*b*). The right panels show views of vertical cross sections corresponding to the left panels (*b–f*). Nuclei were labelled with DAPI (blue), cell membranes were stained by anti-Dlg antibody (red). Imaging of prepared samples was conducted by a Leica confocal microscope (Leica SP5). See the electronic supplementary material for detailed genotypes.

The gene *spz* encodes the *Drosophila* ligand for Toll receptor that activates the Toll pathway in embryonic development and the innate immune response [8,9,70]. As JNK signalling triggers Toll pathway activation and depletion of Toll receptor suppresses JNK-induced cell death, we postulated that JNK signalling might operate upstream of Toll receptor, for instance, by regulating the transcription of *Spz*. To test this hypothesis, we activated JNK signalling in the adult eye (*GMR*>*Egr*), extracted total mRNA from the heads and performed a quantitative reverse transcription polymerase chain reaction (qRT-PCR) assay. In support of our assumption, *Spz* transcription was upregulated more than three-fold upon JNK activation (figure 8*a*). Spz homologues, referred to as Spz2 to Spz6, have been identified in the *Drosophila* genome by an iterative searching method [71]. They share a characteristic intron–exon structure with the prototype *spz* gene, and could execute a similar or redundant function as Spz by binding to the Toll receptors [71]. Intriguingly, activation of JNK signalling is able to upregulate the transcription of all five Spz homologues, with Spz2 level increased by more than 20-fold, as analysed by qRT-PCR assay (figure 8*a*).

To confirm the qRT-PCR data *in vivo*, we examined the transcription of *Spz6* with a *Spz6*-*GFP* reporter strain [72]. Elevated *Spz6*-*GFP* expression was noted posterior to MF in third-instar larval eye discs when JNK signalling was activated by ectopic expression of Egr, dTAK1 or Hep, driven by *GMR*-*GAL4* whose expression pattern was visualized by mRFP (figure 8*b–f*), suggesting that gain of JNK signalling could induce Toll pathway activation through transcriptional upregulation of Spz family ligands. Consistent with our observation that induction of the Toll pathway by activated JNK signalling is caspase independent (electronic supplementary material, figure S9*a–c*), the increased *Spz6-GFP* expression was not suppressed by expressing the caspases inhibitor P35 (electronic supplementary material, figure S9*d*,*e*), but was suppressed by the expression of Bsk^DN^ that served as a positive control (electronic supplementary material, figure S9*f*).

While we found that *Spz6*-*GFP* expression is upregulated by JNK signalling in third-instar larval eye discs (figure 8*b–f*), we are not sure this activation is cell autonomous or non-cell autonomous. As previous study has shown that Spz ligands produced by haemocytes could induce the activation of the Toll/NF-κB pathway in the fat body non-cell autonomously [73], we wondered whether the *Spz6*-*GFP* expressing cells are haemocytes attached to the eye disc. To test this possibility, we performed antibody staining against the haemocyte marker NimC1 [74]. We observed numerous haemocytes associated with control eye discs (electronic supplementary material, figure S9*g*), yet the haemocyte number remained unchanged upon activation of JNK signalling by *GMR*>*Egr* or *GMR*>*dTAK1* (electronic supplementary material, figure S9*h* and *i*). In the meantime, we found that the region attached by haemocytes does not overlap with that of *Spz6*-*GFP* expression (figure 8*d–f* and electronic supplementary material, figure S9*g–i*). Together, these data suggest that *Spz6*-*GFP* expressing cells induced by JNK signalling are not haemocytes.

Anatomically, the eye imaginal disc, with the sac-like two-layered structure, comprises a columnar cell monolayer (named disc proper, DP) covered by a squamous epithelium known as the peripodial membrane (PM) [75]. As the communications between the two distinct cell layers are important for concerted growth and patterning of the disc during development [76,77], we examined the vertical cross section of eye imaginal discs to investigate whether the *Spz6*-*GFP* expressing cells are DP or PM cells. Indeed, we observed two opposing cell layers, PM (P) and DP (D), in the control discs (figure 8*c*), with *GMR-GAL4* expression, marked by mFRP, specifically located within the DP cells (figure 8*b*). When JNK signalling was activated by ectopic expression of Egr, dTAK1 or Hep driven by *GMR-GAL4* in the DP cells, *Spz6*-*GFP* expression was significantly increased in the PM cells (figure 8*d–f*). These results suggest that Spz ligands are induced by activated JNK signalling in a non-cell-autonomous manner in eye discs.

### 2.9. Gain of Toll signalling aggravates Egr-induced cell death in eye development

**Figure 9.**
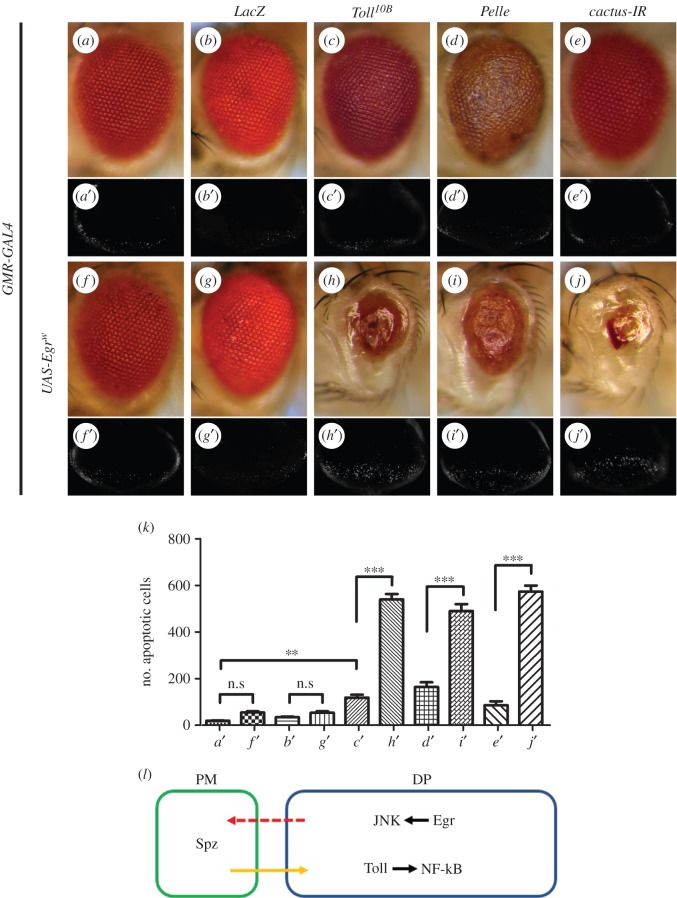
Gain of Toll signalling enhances *GMR*>*Egr*-induced cell death. Light micrographs of *Drosophila* adult eyes (*a–j*) and fluorescence micrographs of third-instar larval eye discs (*a*′–*j*′) are shown. Compared with control (*a* and *a*′), expression of Toll^10B^, Pelle, a *cactus* RNAi or a weak Egr line (Egr^W^) promotes little or mild cell death in eye discs and adult eyes (*c–f* and *c*′–*f*′). Co-expression of Egr^W^ with Toll^10B^, Pelle or a *cactus* RNAi displays synergistic enhancement of cell death in eye discs and adult eyes (*h*–*j* and *h*′–*j*′). As a negative control, expression of LacZ neither triggers cell death by itself (*b* and *b*′) nor aggravates Egr^W^-induced cell death (*g* and *g*′). (*k*) Statistical analysis of cell death in eye discs shown in (*a*′–*j*′). Average number of dying cells labelled by AO staining are counted. Error bars indicate standard deviation. One-way ANOVA with Bonferroni multiple comparison test was used to compute *p*-values, significance was indicated with asterisks (**p < 0.01, ***p < 0.001, *n* = 10 in each group); n.s., not significant. (*l*) A model for the role of the Spz/Toll/NF-κB pathway in modulating Egr-triggered JNK-mediated cell death. See the electronic supplementary material for detailed genotypes.

To further characterize the role of the Toll pathway in cell death, we upregulated Toll signalling in the developing eye by expressing Toll, Pelle, Dorsal, Dif, or an RNAi of *cactus*, the unique *Drosophila* orthologue of IκB that operates as a negative regulator of the Toll pathway [78]. Compared with the controls or expression of LacZ (figure 9*a*,*a*′,*b*,*b*′ and *k*), enhanced Toll signalling results in mild but significant cell death in the eye discs (figure 9*c*′–*e*′,*k* and electronic supplementary material, figure S10*a–d*) and produces rough eyes in adults (figure 9*c–e* and electronic supplementary material, figure S10*e* and *i*), suggesting that activation of the Toll pathway is sufficient to induce cell death. To confirm the activation of Toll signalling, we expressed Toll, Pelle and *cactus-IR* in the fat body, and monitored the expression of *Drs* by *Drs*-*GFP* [64], the recognized Toll pathway reporter [10,13]. We found that the activation of Toll signalling induced by Toll^10B^ (an activated form of Toll) or *cactus-IR* is much stronger than that induced by Pelle (electronic supplementary material, figure S11). We next questioned whether elevated Toll signalling could boost Egr-induced cell death. For this purpose, a weak *UAS*-*Egr* line (*UAS*-*Egr^w^*) [29] was expressed in the eye, which caused limited cell death in third-instar eye discs and adult eyes (figure 9*f*,*f*′,*k*). A synergistic enhancement in cell death and eye size reduction was observed when Egr^w^ was co-expressed with Toll, Pelle or the *cactus* RNAi, but not with LacZ (figure 9*g–j*,*g*′–*j*′,*k*), suggesting that gain of Toll signalling exacerbates Egr-triggered cell death.

### 2.10. Toll signalling-triggered cell death is independent of JNK, caspase or necroptosis

While the Toll/NF-κB pathway is able to promote cell death, the underlying mechanisms remains largely unknown. We found that expression of Bsk^DN^, or Puckered (Puc) encoding a JNK phosphatase that negatively regulates JNK kinase activity [63], exhibits no suppressive effect on the rough eye phenotypes induced by Dorsal or Dif expression (electronic supplementary material, figure S10*e–l*), implying that the JNK pathway is not involved in Toll pathway-induced cell death.

Our previous data have implied that the Toll signalling pathway is not required for caspases-mediated cell death (electronic supplementary material, figure S5). To further investigate the relationship between Toll pathway-induced cell death and caspases, we firstly checked antibody staining for the activated form of Caspase-3 (CC-3), a read-out of the initiator caspase Dronc (*Drosophila* caspase-9) activity [79], and found that gain of Toll signalling by over-expressing Dorsal or Dif did not result in increased caspases activity (electronic supplementary material, figure S12*a–d*). Consistently, we also observed that the rough and small eye phenotypes induced by Dorsal or Dif expression remain unaffected by blocking caspase signalling (electronic supplementary material, figure S12*e–h*,*k–n*). Together, these data indicated that the Toll pathway triggers caspase-independent cell death.

Necroptosis/programmed necrosis, which is a RIP1/RIP3-dependent caspase-independent programmed necrotic cell death, plays essential roles in development [80–84], and has been implicated in a variety of pathological progresses, including tumourigenesis, metastasis, inflammation and liver diseases [85–87]. To investigate whether cell death induced by the Toll/NF-κB pathway is mediated by necroptosis, we depleted dTRAF2, the key component of necroptosis machinery [88,89]. We found that mutation or RNAi-mediated inactivation of *dTRAF2* has no effect on the rough eye phenotypes of *GMR*>*Dorsal* or *GMR*>*Dif* (electronic supplementary material, figure S12*i*,*j*,*o*,*p*), indicating that Toll/NF-κB pathway-triggered cell death is independent of necroptosis.

## 3. Discussion

The Toll pathway has been implicated in embryonic dorsal–ventral patterning and innate immunity in *Drosophila*. In this study, we demonstrate that the Toll pathway modulates JNK-mediated cell death *in vivo*, adding a distinct but vital role to this well-characterized signalling pathway, and further show that JNK signalling triggers Toll pathway activation through transcriptional upregulation of Spz family members encoding the ligands for Toll receptor in a non-cell-autonomous manner (figure 9*l*). In the light of their conserved function from fly to human, the implication of the *Drosophila* Toll pathway in JNK signalling-mediated cell death shall provide a novel connection for the crosstalk between IL-1R and JNK signalling in cell death in mammals.

We found that loss of Spz, either by mutation or RNAi inactivation, failed to suppress *GMR*-Egr-triggered cell death (data not shown), suggest other Spz family ligands may play redundant function in modulating cell death. Consistent with this interpretation, Spz2 was shown to play redundant function with other Spz ligands and regulate cell death in CNS [90]. As there is no mutant allele available for other *spz* genes at the moment, it is not feasible to determine whether all or a specific set of Spz molecules are required for JNK-induced Toll pathway activation. Further investigation will be required to clarify this matter.

Recent study reported that the Toll pathway could be activated by danger signals released by apoptosis-deficient cells in a non-cell-autonomous manner [91]. Consistently, we found that elevated JNK signalling triggers Toll pathway activation independent of caspase-mediated apoptosis (electronic supplementary material, figure S9*a–f*). Furthermore, we provide compelling evidence for a molecular mechanism of this activation: activated JNK signalling in the columnar disc (DP) cells induces elevated expression of *Spz* family ligands in the PM cells (figure 8*b–f*), which in turn activates the Toll/NF-κB pathway in DP cells (figure 9*l*). Thus, our data reveal a functional interplay between the DP cells and the PM cells in eye discs. However, the mechanism by which JNK signalling triggers the expression of Spz ligands non-cell autonomously remains elusive.

A recent study suggested that the Toll/NF-κB pathway promotes JNK-independent cell death by upregulating the expression of pro-apoptotic gene *rpr* in the loser cells of wing discs during cell competition [92]. However, we have provided evidence indicating that Toll pathway-mediated cell death is mostly caspase-independent: (i) loss of Toll signalling does not suppress *Sd*>*Hep* induced transcriptional upregulation of *hid* in the wing disc (figure 5*o–r*); (ii) loss of Toll signalling does not affect *GMR*>*Hid* induced small eye phenotype (electronic supplementary material, figure S5); (iii) *GMR*>*Dorsal* or *GMR*>*Dif*-induced cell death in third-instar larval eye discs (electronic supplementary material, figure S10*a–d*) is independent of caspase activation (electronic supplementary material, figure S12*a–c*) and (iv) *GMR*>*Dorsal* and *GMR*>*Dif*-induced small eye phenotypes are not suppressed by blocking caspase signalling (electronic supplementary material, figure S12*e–h*,*k–n*). In addition, we found that Toll pathway-triggered cell death in the eye is independent of JNK or necroptosis (electronic supplementary material, figure S10*e–l*; S12*i*, *j*, *o*, *p*). Thus, activated Toll signalling may promote cell death via distinct mechanisms in a context-dependent manner.

## 4. Material and methods

### 4.1. Fly strains

Flies were kept on a cornmeal and agar medium at 25°C according to standard protocols. *Drosophila* strains used include: *Toll^r3^*, *Toll^r4^*, *Dif^1^*, *spz^2^*, *spz^3^*, *imd^1^, relish^E38^*, *UAS-Toll-IR* (31044, 41477 and 35628), *UAS-pelle-IR* (34733 and 35577), *Spz6*-*GFP* (23305), *UAS*-*mGFP* (32197), *UAS-mRFP* (32218), *UAS-Dorsal* (9319), *UAS*-*Dif* (22201), *UAS*-*dJun* (7216), *UAS*-*dFos* (7213), *UAS*-*dFoxO*, *UAS-DIAP1*, *UAS-Bsk*, *Df(3L)H99*, *Cg*-*GAL4* (7011), *Tub*-*GAL80^ts^* (7017), *Drs*-*GFP Dipt*-*LacZ* (55707), *yw^1118^ hs*-*Flp*; *act*>y + >*GAL4 UAS*-*GFP*, these and the deficiency kit were obtained from Bloomington *Drosophila* stock centre. *UAS-puc-IR* (3018 and 3019), *UAS-dorsal-IR* (45996 and 45998), *UAS*-*Dif-IR* (30578 and 30579) and *UAS-relish-IR* (49413 and 49414) were obtained from Vienna *Drosophila* RNAi centre. *UAS-tube-IR* (105520R1 and 10520R3), *UAS-imd-IR* (5576R1 and 5576R2), *UAS-cactus-IR* (5848R1 and 5848R3) and *UAS-bsk-IR* (5680R2) were obtained from Fly Stocks of National Institute of Genetics (NIG). *GMR*-*GAL4* [56], *Sd*-*GAL4*, *ptc*-*GAL4* [57], *UAS*-*Toll^10B^* [93], *Toll*^*EP(3)1051*^ [94], *UAS*-*Egr*, *UAS*-*Egr^W^* [29], *UAS*-*Egr^KB^* [30], *UAS*-*Bsk^DN^* [31], *UAS-Puc*, *sev-GAL4*, *UAS*-*dTAK1*, *UAS*-*Hep^CA^*, *UAS*-*Hep*, *UAS*-*Grim^M146^*, *UAS-Hid^M137^*, *UAS-Imd.SN^F32^*, *UAS*-*Dronc^DN^* [29], *UAS*-*P35*, *UAS*-*GFP* [41,42,52], *hid*-*LacZ* [40], *dTRAF2^EX1.1^* and *UAS-dTRAF2-IR* were previously described.

### 4.2. Acridine orange staining

Eye and wing discs were dissected from third-instar larvae in 0.3% PBST (phosphate-buffered saline (PBS) + 0.3% Triton X-100) and incubated in 1 × 10^−5^ M AO for 5 min at room temperature prior to imaging as described [40].

### 4.3. X-gal staining

Wing discs were dissected from third-instar larvae in 0.1% PBST (PBS + 0.1% Triton X-100) and stained for ß-galactosidase activity as described [95].

### 4.4. Immunohistochemistry

Fat body and eye discs dissected from third-instar larvae were collected in cold PBS, and fixed in 4% paraformaldehyde. After fixation, samples were washed three times in 0.3% PBST, blocked in 10% horse serum and stained with primary antibody overnight at 4°C. Samples were washed as previously described and subjected to secondary antibodies for 2 h. Primary antibodies used included mouse anti-dorsal (1 : 100, Cell Signaling Technology), mouse anti-Dlg (1 : 200, DSHB), mouse anti-NimC1 (1 : 200, kind gift of I. Ando) and rabbit anti-Cleaved Caspase-3 (1 : 200, Cell Signaling Technology). Secondary antibodies used were anti-mouse-Cy3 (1 : 1000, Jackson Immuno Research) and anti-rabbit- Cy3 (1 : 1000, Cell Signaling Technology).

### 4.5. Quantitative reverse transcription polymerase chain reaction

Thirty adult heads were collected from freshly eclosed flies of indicated genotypes. Total RNA was isolated using TRIzol (Invitrogen), and RT-PCR was performed as previously described [96]. Primers for *Rp49* and *spz1–6* were kindly provided by Dr Ketu Mishra at Yale University.

## 5. Statistics

Results are presented as bar graphs or scatter plots created using GraphPad Prism 6.0. A combination of unpaired *t*-test and one-way ANOVA with Bonferroni's multiple comparison test was used to calculate statistical significance; *p*-values are included in the relevant figure legends.

## Supplementary Material

Supplementary Information
