# Supplementary material for: Toll pathway modulates TNF-induced JNK-dependent cell death in Drosophila
Source: Open Biol. 2015 Jul 22;5(7):140171. doi: 10.1098/rsob.140171 (PMC4632500; doi:10.1098/rsob.140171)

Supplementary Information

Figure S1. Inactivation of Imd pathway cannot suppress *GMR>Egr*-triggered small eye phenotype.

Light micrographs showing *Drosophila* adult eyes. The *GMR>Egr*-induced small eye phenotype (**a**, **d** and **g**) is not suppressed by knocking-down *imd* (**b**) or *relish* (**c**), or deleting both copies of endogenous *imd* (**f**) or *relish* (**i**), or expressing LacZ (**e** and **h**).

Figure S2. Ectopic expression of Grim induces loss-of-ACV phenotype.

(**a** and **b**) Light micrographs showing *Drosophila* adult wings. Compared with the control (**a**), limited expression of Grim driven by *ptc-GAL4* with *Tub-GAL80*^{ts} produces the loss-of-ACV phenotype (**b**). The lower panels show high magnification views of the boxed areas in the top panels. (**c**) Quantification of the ACV phenotype as shown in figures **a** and **b** (n=20, for each genotype). Error bars indicates standard deviation. Unpaired t test was used to calculate statistical significance, indicated with asterisks (*** $P<0.001$).

Figure S3. Expression of Hep triggers cell death in wing discs.

(**a** and **b**) Fluorescence micrographs of third instar larval wing discs are shown. Compared with the control (**a**), expression of Hep driven by *Sd-GAL4* induces evident cell death in larval wing discs indicated by AO staining (**b**). (**c**) Statistical analysis of cell death in wing discs (n=10) as shown in figures **a** and **b.** Error bars indicate standard deviation. Unpaired t test was used to calculate statistical significance, indicated with asterisks (*** $P<0.001$).

Figure S4. Loss of Toll signaling suppresses JNK-mediated cell death.

Light micrographs of *Drosophila* adult wings (**a**-**e**) or third instar larval wing discs (**g**-**k**) are shown. Compared with *Sd-GAL4* control (**a** and **g**), ectopic expression of Hep driven by *Sd-GAL4* results in a dramatic size reduction in adult wings (**b**) and larval wing discs (**h**), both of which are suppressed significantly by expressing Bsk^{DN} (**d** and

j) or a *dorsal* RNAi (**e** and **k**), but not that of GFP (**c** and **i**). (**f**) Statistical analysis of the adult wing size as shown in figures **a-e**. One-way ANOVA with Bonferroni multiple comparison test was used to compute *P*-values, significance was indicated with asterisks (*** P<0.001). ns stands for not significant.

Figure S5. Inactivation of Toll pathway cannot suppress *GMR>Hid*-induced small eye phenotype.

Light micrographs showing *Drosophila* adult eyes. The *GMR>Hid*-triggered small eye phenotype (**a**) is not suppressed by knocking-down *dorsal* (**b**) or *Dif* (**c**).

Figure S6. Egr fails to induce *Dipt* expression in fat body cells.

Light micrographs of *Drosophila* third instar larval fat body are shown. *Dipt* expression in third instar larval fat body cells (**a**) cannot be activated by ectopic expression of Egr under the control of *Cg-GAL4* (**b**), but triggered by expression of Imd (**c**), detected by X-gal staining of *Dipt*-LacZ.

Figure S7. Gain of JNK signaling induces dorsal expression in eye discs.

Fluorescent microscope images showing eye discs dissected from third-instar larvae stained with anti-Dorsal (red). Compared with the control (**a**), the level of endogenous Dorsal was up-regulated by expressing dTAK1 (**b**). **a'-a'''** and **b'-b'''** show high magnification views of the boxed areas in **a** and **b**, respectively. White arrows indicate the regions where Dorsal staining overlaps with the nucleus. Nuclei were labeled with DAPI (blue). Imaging of prepared sample was conducted by a Leica confocal microscope (Leica SP5).

Figure S8. Gain of JNK signaling promotes dorsal nuclear-localization in fat body cells.

Fluorescent microscope images showing fat body cells dissected from third-instar larval stained with anti-dorsal (red). Nuclei were marked with DAPI (blue), cell membranes

were tagged by GFP (green). Compared with the control (**a**), expression of Egr (**b**) or RNAi inactivation of *puc* (**c**) in fat body promotes the nuclear accumulation of Dorsal. (**d**) Quantification of the nuclear/cytoplasmic ratio of Dorsal in fat body cells shown in **a**-**c**. Anti-Dorsal intensities were measured in pixels using Image J. Error bars showed standard deviation from measurement of at least 15 cells for each genotype. One-way ANOVA with Bonferroni multiple comparison test was used to calculate statistical significance, indicated with asterisks (*** P<0.001)

Figure S9. Blocking caspases does not affect JNK-triggered Toll activation. Fluorescence micrographs of third instar larvae (**a** and **b**), dissected fat body cells stained with anti-dorsal (red) (**c**) and eye discs (**d**-**i**) are shown. Nuclei were labeled with DAPI (blue). The up-regulated *Drs-GFP* expression and the nuclear accumulation of endogenous Dorsal induced by activated JNK signaling were caspase-independent (**a**-**c**). dTAK1+P35-expressing clones were tagged by GFP (green) and induced for 1h by heat shock at 37°C and recovered for 24h at 25°C. (**d**-**f**) The up-regulated expression of *Spz6-GFP* triggered by ectopic dTAK1 (**d**) can be suppressed by expressing Bsk^{DN} (**f**), but not by P35 (**e**). Cell membranes were stained by anti-Dlg antibody (red). (**g**-**i**) Compared with the control (**g**), the number of hemocytes attached to eye discs is not significantly changed by ectopic expression of Egr (**h**) or dTAK1 (**i**) driven by *GMR-GAL4*. Hemocytes were stained by anti-NimC1 antibody. Images **g**-**i** were taken in a Zeiss LSM780.

Figure S10. JNK signaling is not involved in Toll/NF-κB triggered cell death. (**a**-**c**) Fluorescence micrographs of third instar larval eye discs are shown. Compared with the control (**a**), both *GMR>Dorsal* and *GMR>Dif* induce cell death in larval eye discs (**b** and **c**). (**d**) Statistic analysis of cell death in eye discs shown in (**a**-**c**). Average number of dying cells labelled by AO staining are counted. Error bars indicates standard deviation. One-way ANOVA with Bonferroni multiple comparison test was used to compute *P*-values, significance was indicated with asterisks (*** P<0.001, n=10). (**e**-**l**) Light micrographs showing *Drosophila* adult eyes. Expression of Dorsal (**e**) or Dif (**i**)

under the control of the *GMR* promoter triggers cell death and generates rough eyes with reduced size, which is not suppressed by expressing of LacZ (**f** and **j**), Bsk^{DN} (**g** and **k**) or Puc (**h** and **l**).

Figure S11. The expression of *Drs-GFP* is induced by gain of Toll signaling in the fat body.

Fluorescence micrographs of third instar larvae are shown. The *Drs-GFP* is significantly up-regulated by gain of Toll signaling in the fat body.

Figure S12. Toll/NF-κB triggered cell death is independent of caspase or necroptosis.

(**a**-**c**) Fluorescence micrographs of third instar larval eye discs are shown. Compared with the control (**a**), no increased caspase activity is observed in *GMR*>*Dorsal* or *GMR*>*Dif* eye discs (**b** and **c**). (**d**) Statistic analysis of caspase activation in eye discs shown in (**a**-**c**). Average number of cells labelled by anti-CC-3 staining are counted. Error bars indicates standard deviation. One-way ANOVA with Bonferroni multiple comparison test was used to compute *P*-values, significance was indicated with asterisks (*** $P<0.001$, n=10). (**e**-**p**) Light micrographs showing *Drosophila* adult eyes. The rough and small eye phenotypes of *GMR>Dorsal* (**e**) and *GMR>Dif* (**k**) are not suppressed by blocking caspases signaling (**f**-**h** and **l**-**n**) or knocking-down dTRAF2 (**i**, **j**, **o** and **p**).

<u>Detailed Genotypes</u>

Figure 1

(b) *GMR-GAL4*/+

(c) *UAS-Egr*/+; *GMR-GAL4*/+

(d) *UAS-Egr*/+; *GMR-GAL4*/*Df(3R)ED6232*

(e) *UAS-Egr*/+; *GMR-GAL4*/*Df(3R)BSC496*

(f) *UAS-Egr*/+; *GMR-GAL4*/*Df(3R)BSC524*

(g) *UAS-Egr*/+; *GMR-GAL4*/*Df(3R)ED6255*

(h) *UAS-Egr*/*Df(2L)BSC294*; *GMR-GAL4*/+

(i) *UAS-Egr*/+; *GMR-GAL4*/*Toll*^{r3}

Figure 2

(c) *GMR*-GAL4/+

(d) *UAS-Egr*/+; *GMR-GAL4*/+

(e) *UAS-Egr*/+; *GMR-GAL4*/*UAS*-LacZ

(f) *UAS-Egr*/*imd*^{1}; *GMR-GAL4*/+

(g) *UAS-Egr*/*Dif*^{1}; *GMR-GAL4*/+

(h) *UAS-Egr*/+; *GMR-GAL4*/*UAS-Toll-IR* (BL31044)

(i) *UAS-Egr*/+; *GMR-GAL4*/*UAS-tube-IR* (NIG105520R3)

(j) *UAS-Egr*/+; *GMR-GAL4*/*UAS-pelle-IR* (BL34733)

(k) *UAS-Egr*/+; *GMR-GAL4*/*UAS-dorsal-IR* (V45998)

(l) *UAS-Egr*/+; *GMR-GAL4*/*UAS-Dif-IR* (V30579)

Figure 3

(a) *sev-GAL4*/*UAS-dTAK1*

(b) *sev-GAL4*/*UAS-dTAK1*; *UAS-LacZ*/+

(c) *sev-GAL4*/*UAS-dTAK1*; *UAS-bsk-IR*/+

(d) *sev-GAL4*/*UAS-dTAK1*; *UAS-pelle-IR* (BL34733)/+

(e) *sev-GAL4*/*UAS-dTAK1*; *UAS-dorsal-IR* (V45998)/+

(f) *GMR-GAL4 UAS-Hep*^{CA}/+

(g) *GMR-GAL4 UAS-Hep*^{CA}/*UAS-LacZ*

(h) *GMR-GAL4 UAS-Hep*^{CA}/*UAS-bsk-IR*

(i) *GMR-GAL4 UAS-Hep*^{CA}/*UAS-pelle-IR* (BL34733)

(j) *GMR-GAL4 UAS-Hep*^{CA}/*UAS-dorsal-IR* (V45998)

(k) *GMR-GAL4*/*UAS-Bsk*

(l) *GMR-GAL4*/*UAS-Bsk*; *UAS-GFP*/+

(m) *GMR-GAL4*/*UAS-Bsk*; *UAS-pelle-IR* (BL34733)/+

(n) *GMR-GAL4*/*UAS-Bsk*; *UAS-dorsal-IR* (V45998)/+

(o) *GMR-GAL4*/*UAS-Bsk*; *UAS-Dif-IR* (V30579)/+

Figure 4

(a) *ptc-GAL4*/+

(b) *ptc-GAL4*/*UAS-Egr*^{W}

(c) *ptc-GAL4*/*UAS-Egr*^{W}; *UAS-LacZ*/+

(d) *ptc-GAL4*/*UAS-Egr*^{W}; *UAS-Bsk*^{DN}/+

(e) *ptc-GAL4*/*UAS-Egr*^{W}; *UAS-Toll-IR* (BL31044)/+

(f) *ptc-GAL4*/*UAS-Egr*^{W}; *UAS-dorsal-IR* (V45998)/+

(g) *ptc-GAL4*/*UAS-Egr*^{W}; *UAS-Dif-IR* (V30579)/+

(h) *ptc-GAL4*/*UAS-Hep*

(i) *ptc-GAL4*/*UAS-Hep*; *UAS-LacZ*/+

(j) *ptc-GAL4*/*UAS-Hep*; *UAS-Bsk*^{DN}/+

(k) *ptc-GAL4*/*UAS-Hep*; *UAS-Toll-IR* (BL31044)/+

(l) *ptc-GAL4*/*UAS-Hep*; *UAS-dorsal-IR* (V45998)/+

(m) *ptc-GAL4*/+; *Toll*$^{EP(3)1051}$/+

Figure 5

(a) *Sd-GAL4*/+

(b) *Sd-GAL4*/+; *UAS-dJun*/+

(c) *Sd-GAL4*/+; *UAS-dFos*/+

(d) *Sd-GAL4*/+; *UAS-dFoxO*/+

(e) *Sd-GAL4*/+; *UAS-dFoxO*/+; *UAS-GFP*/+

(f) *Sd-GAL4*/+; *UAS-dFoxO*/+; *UAS-Toll-IR* (BL31044)/+

(g) *Sd-GAL4*/+; *UAS-dFoxO*/+; *UAS-dorsal-IR* (V45998)/+

(h) *Sd-GAL4*/+; *UAS-dFoxO*/+; *UAS-Dif-IR* (V30579)/+

(j) *Sd-GAL4*/+; *UAS-Hep*/+

(k) *Sd-GAL4*/+; *UAS-Hep*/+; *H99*/+

(l) *Sd-GAL4*/+; *UAS-Hep*/+; *UAS-dIAP1*/+

(m) *Sd-GAL4*/+; *UAS-Hep*/+; *UAS-Dronc*^{DN}/+

(o) *Sd-GAL4*/+; *hid-LacZ*/+

(p) *Sd-GAL4*/+; *UAS-Hep*/+; *hid-LacZ*/+

(q) *Sd-GAL4*/+; *UAS-Hep*/+; *hid-LacZ*/*UAS-dorsal-IR* (V45998)

(r) *Sd-GAL4*/+; *UAS-Hep*/+; *hid-LacZ*/*UAS-Dif-IR* (V30579)

Figure 6

(a and h) *ptc-GAL4*/*UAS-puc-IR* (V3108)

(b and i) *ptc-GAL4*/*UAS-puc-IR*; *UAS-LacZ*/+

(c and j) *ptc-GAL4*/*UAS-puc-IR*; *UAS-Bsk*^{DN}/+

(d and k) *ptc-GAL4*/*UAS-puc-IR*; *UAS-Toll-IR* (BL31044)/+

(e and l) *ptc-GAL4*/*UAS-puc-IR*; *UAS-tube-IR* (NIG10520R3)/+

(f and m) *ptc-GAL4*/*UAS-puc-IR*; *UAS-dorsal-IR* (V45998)/+

(g and n) *ptc-GAL4*/*UAS-puc-IR*; *UAS-Dif-IR* (V30579)/+

Figure 7

(a) *Drs-GFP*/+; *Cg-GAL4*/+

(b) *Drs-GFP*/+; *Cg-GAL4*/+; *UAS-dTAK1*/+

(c) *Drs-GFP*/+; *Cg-GAL4*/*UAS-puc-IR* (V3108)

(d) w^{1118} *hs-Flp*/+; *act>y+>GAL4 UAS-GFP*/*UAS-dTAK1*

Figure 8

(b) *Spz6-GFP*/+; *GMR-GAL4*/*UAS-mRFP*

(c) *Spz6-GFP*/+; *GMR-GAL4*/+

(d) *Spz6-GFP*/+; *GMR-GAL4*/*UAS-Egr*^{KB}

(e) *UAS-dTAK1*/*Spz6-GFP*; *GMR-GAL4*/+

(f) *UAS-Hep*^{CA}/*Spz6-GFP*; *GMR-GAL4*/+

Figure 9

(a) *GMR-GAL4*/+

(b) *GMR-GAL4*/*UAS-LacZ*

(c) *UAS-Toll*^{10B}/+; *GMR-GAL4*/+

(d) *GMR-GAL4*/*UAS-Pelle*

(e) *GMR-GAL4*/*UAS-cactus-IR* (NIG5848R3)

(f) *UAS-Egr*^{W}/+; *GMR-GAL4*/+

(g) *UAS-Egr*^{W}/+; *GMR-GAL4*/*UAS-LacZ*

(h) *UAS-Egr*^{W}/*UAS-Toll*^{10B}; *GMR-GAL4*/+

(i) *UAS-Egr*^{W}/+; *GMR-GAL4*/*UAS-Pelle*

(j) *UAS-Egr*^{W}/+; *GMR-GAL4*/*UAS-cactus-IR* (NIG5848R3)

Figure S1

(a) *UAS-Egr*/+; *GMR-GAL4*/+

(b) *UAS-Egr*/*UAS-imd-IR* (NIG5576R2); *GMR-GAL4*/+

(c) *UAS-Egr*/*UAS-rel-IR* (V49413); *GMR-GAL4*/+

(d) *GMR-GAL4 UAS-Egr*^{KB}/+

(e) *GMR-GAL4 UAS-Egr*^{KB}/*UAS-LacZ*

(f) *imd*^{1}/*imd*^{1}; *GMR-GAL4 UAS-Egr*^{KB}/+

(g) *GMR-GAL4 UAS-Egr*/+

(h) *GMR-GAL4 UAS-Egr*/+; *UAS-LacZ*/+

(i) *GMR-GAL4 UAS-Egr*/+; *rel*^{E38}/ *rel*^{E38}

Figure S2

(a) *ptc-GAL4*/+; *Tub-GAL80*^{ts}/+

(b) *ptc-GAL4*/*UAS-Grim*; *Tub-GAL80*^{ts}/+

Figure S3

(a) *Sd-GAL4*/+

(b) *Sd-GAL4*/+; *UAS-Hep*/+

Figure S4

(a and g) *Sd-GAL4*/+

(b and h) *Sd-GAL4*/+; *UAS-Hep*/+

(c and i) *Sd-GAL4*/+; *UAS-Hep*/+; *UAS-GFP*/+

(d and j) *Sd-GAL4*/+; *UAS-Hep*/+; *UAS-Bsk*^{DN}/+

(e and k) *Sd-GAL4*/+; *UAS-Hep*/+; *UAS-dorsal-IR* (V45998)/+

Figure S5

(a) *UAS-Hid*/+; *GMR-GAL4*/+

(b) *UAS-Hid*/+; *GMR-GAL4*/+; *UAS-dorsal-IR* (V45998)/+

(c) *UAS-Hid*/+; *GMR-GAL4*/+; *UAS-Dif-IR* (V30579)/+

Figure S6

(a) *Dipt-LacZ*/+; *Cg-GAL4/*+

(b) *Dipt-LacZ*/+; *Cg-GAL4*/*UAS-Egr*

(c) *Dipt-LacZ*/+; *Cg-GAL4*/+; *UAS-Imd/*+

Figure S7

(a) *GMR-GAL4*/+

(b) *UAS-dTAK1*/+; *GMR-GAL4*/+

Figure S8

(a) *Cg-GAL4*/+

(b) *Cg-GAL4*/*UAS-Egr*^{W}

(c) *Cg-GAL4*/*UAS-puc-IR* (V3108)

Figure S9

(a) *Drs-GFP*/+; *Cg-GAL4*/*UAS-puc-IR* (V3108)

(b) *Drs-GFP*/+; *Cg-GAL4*/*UAS-puc-IR*; *UAS*-P35/+

(c) w^{1118} *hs-Flp*/+; *act>y+>GAL4 UAS-GFP*/*UAS-dTAK1*; *UAS-P35*/+

(d) *UAS-dTAK1*/*Spz6-GFP*; *GMR-GAL4*/+

(e) *UAS-dTAK1*/*Spz6-GFP*; *GMR-GAL4/UAS-P35*

(f) *UAS-dTAK1*/*Spz6-GFP*; *GMR-GAL4*/*UAS-Bsk*^{DN}

(g) *GMR-GAL4*/+

(h) *GMR-GAL4*/*UAS-Egr*^{KB}

(i) *UAS-dTAK1*/+; *GMR-GAL4*/+

Figure S10

(a) *GMR-GAL4*/+

(b and e) *GMR-GAL4*/*UAS-Dorsal*

(c and i) *GMR-GAL4*/*UAS-Dif*

(f) *GMR-GAL4*/*UAS-Dorsal*; *UAS-LacZ*/+

(g) *GMR-GAL4*/*UAS-Dorsal*; *UAS-Bsk*^{DN}/+

(h) *GMR-GAL4*/*UAS-Dorsal*; *UAS-Puc*/+

(j) *GMR-GAL4*/*UAS-Dif*; *UAS-LacZ*/+

(k) *GMR-GAL4*/*UAS-Dif*; *UAS-Bsk*^{DN}/+

(l) *GMR-GAL4*/*UAS-Dif*; *UAS-Puc*/+

Figure S11

(a) *Drs-GFP*/+; *Cg-GAL4*/*UAS-Toll*^{10B}, *Drs-GFP*/+; *Cg-GAL4*/+; *UAS-Pelle*/+, *Drs-GFP*/+; *Cg-GAL4*/+; *UAS-cactus-IR* (NIG5848R3)/+, *Drs-GFP*/+; *Cg-GAL4*/+ (from left to right)

Figure S12

(a) *GMR-GAL4*/+

(b and e) *GMR-GAL4*/*UAS-Dorsal*

(c and k) *GMR-GAL4*/*UAS-Dif*

(f) *GMR-GAL4*/*UAS-Dorsal*; *H99*/+

(g) *GMR-GAL4*/*UAS-Dorsal*; *UAS-Dronc*^{DN}/+

(h) *GMR-GAL4*/*UAS-Dorsal*; *UAS*-P35/+

(i) *dTRAF2*$^{EX1.1}$/+; *GMR-GAL4*/*UAS-Dorsal*

(j) *GMR-GAL4 UAS-Dorsal*/*UAS-dTRAF2-IR*

(l) *GMR-GAL4*/*UAS-Dif*; *H99*/+

(m) *GMR-GAL4*/*UAS-Dif*; *UAS-Dronc*^{DN}/+

(n) *GMR-GAL4*/*UAS-Dif*; *UAS-P35*/+

(o) *dTRAF2*$^{EX1.1}$/+; *GMR-GAL4*/*UAS-Dif*

(p) *GMR-GAL4 UAS-Dif*/*UAS-dTRAF2-IR*

Wu et al., Figure S1

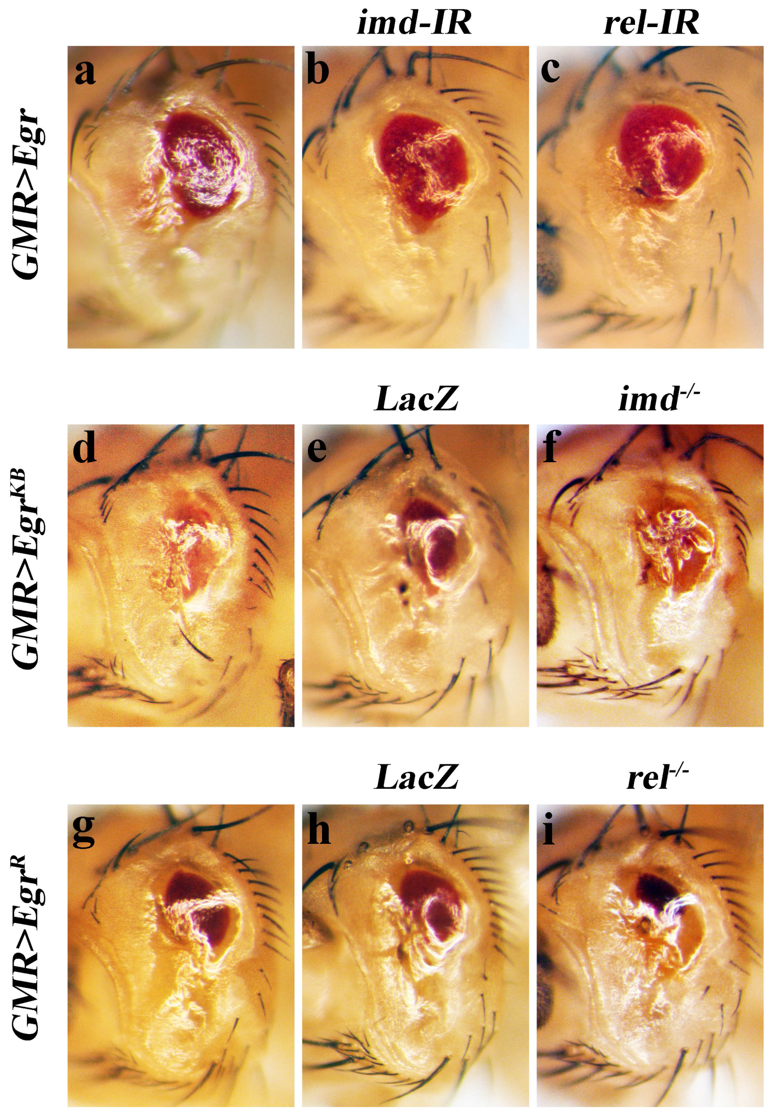

Wu et al., Figure S2

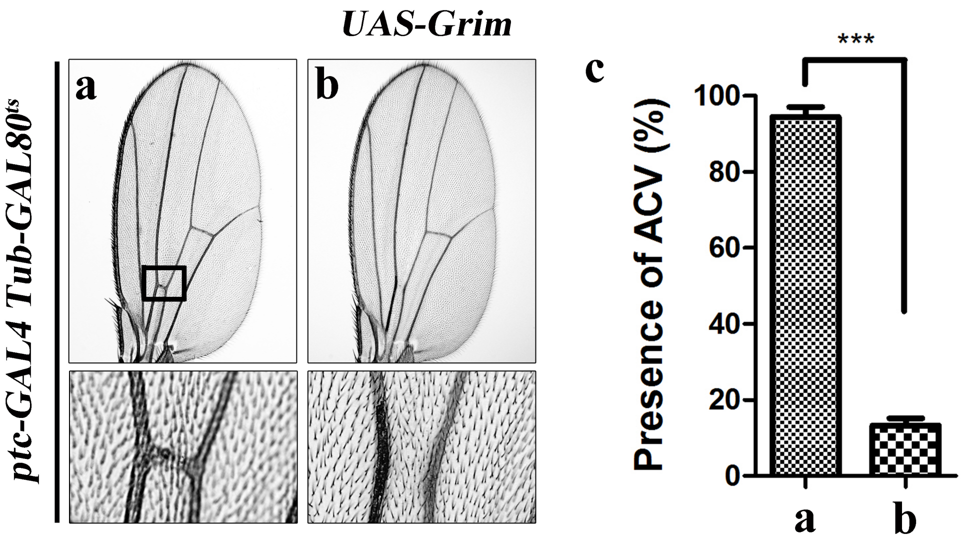

Wu et al., Figure S3

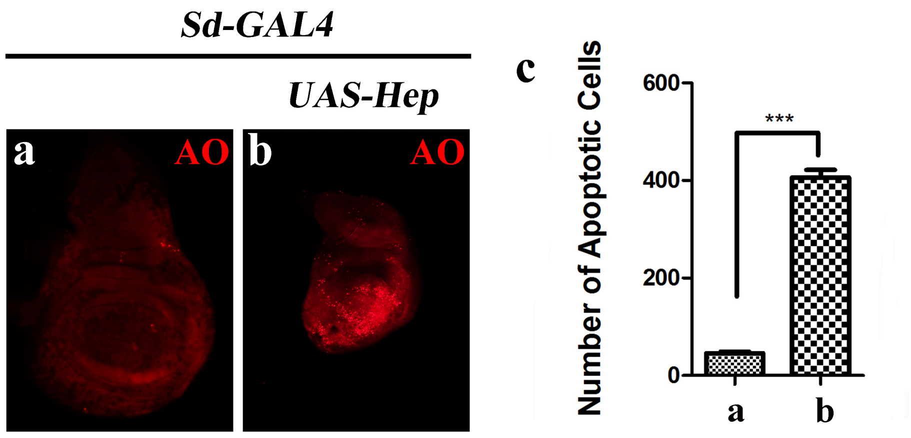

Wu et al., Figure S4

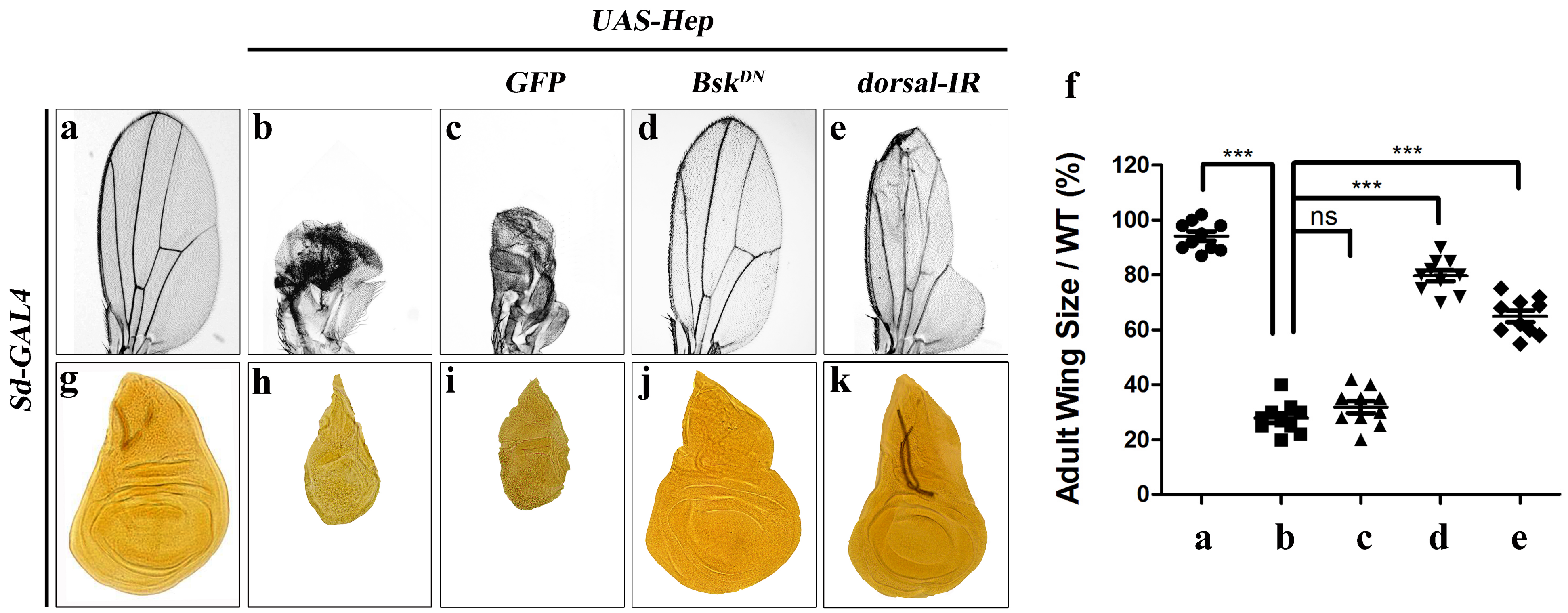

Wu et al., Figure S5

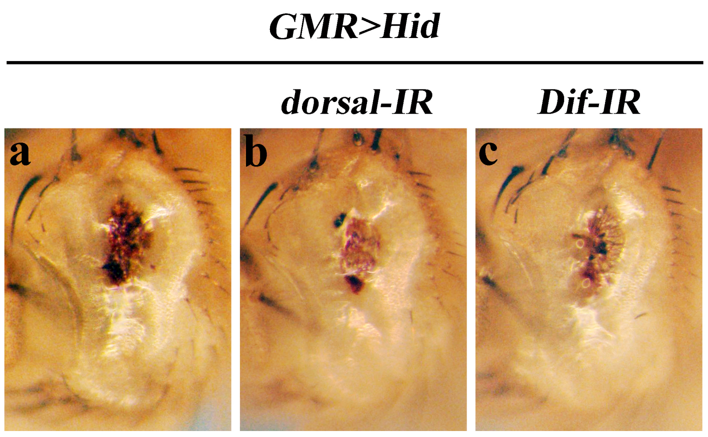

Wu et al., Figure S6

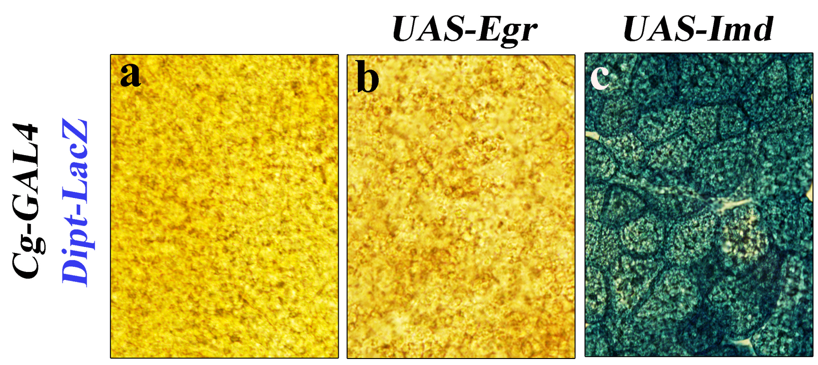

Wu et al., Figure S7

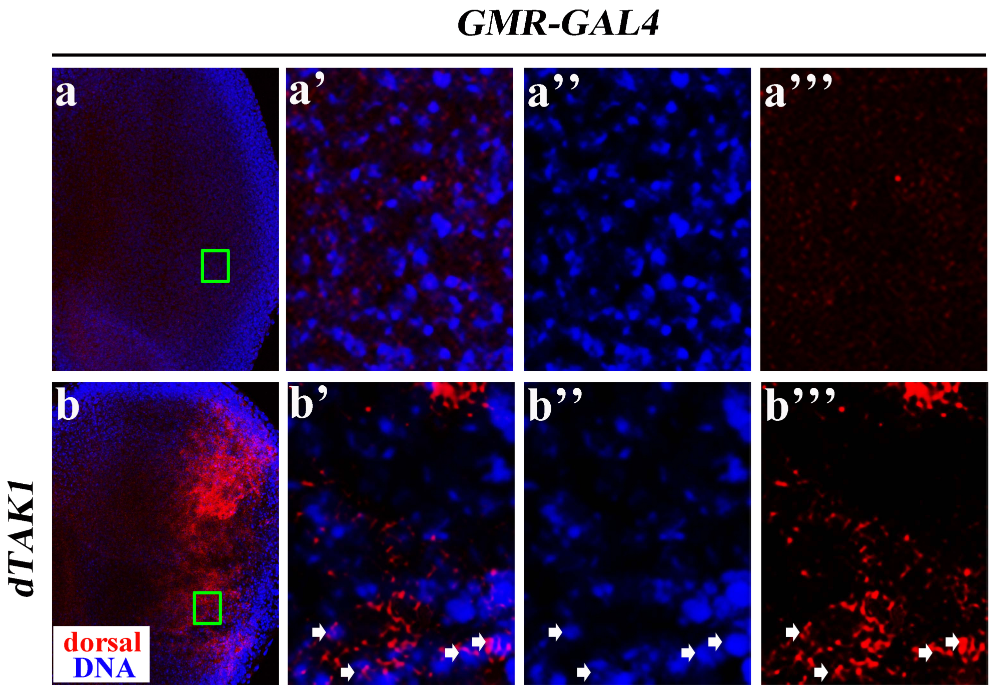

Wu et al., Figure S8

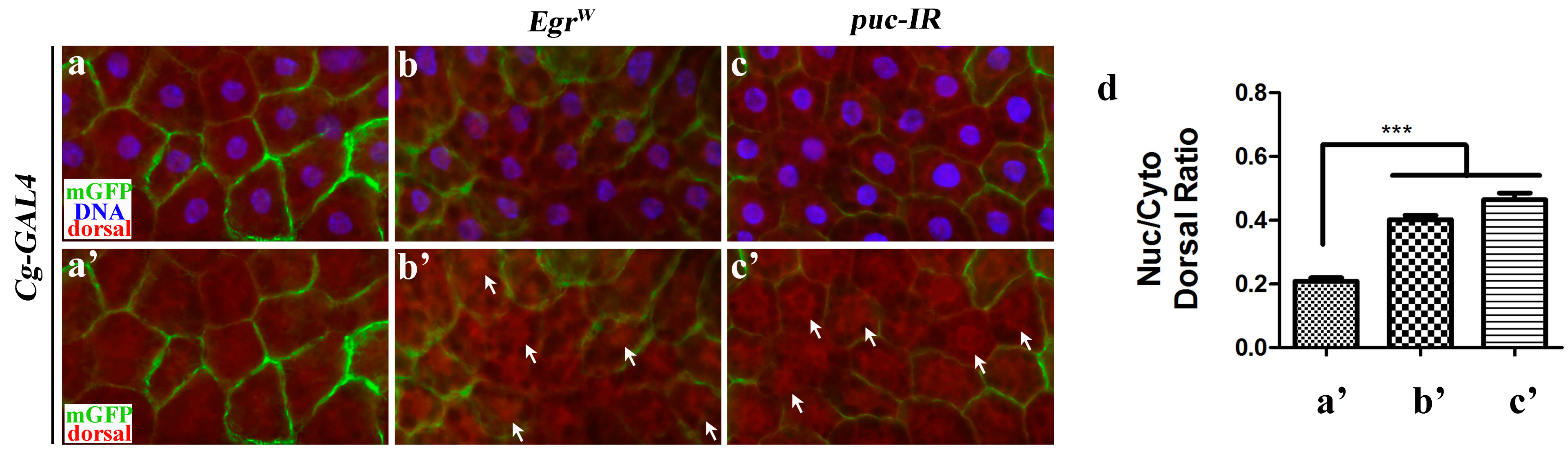

Wu et al., Figure S9

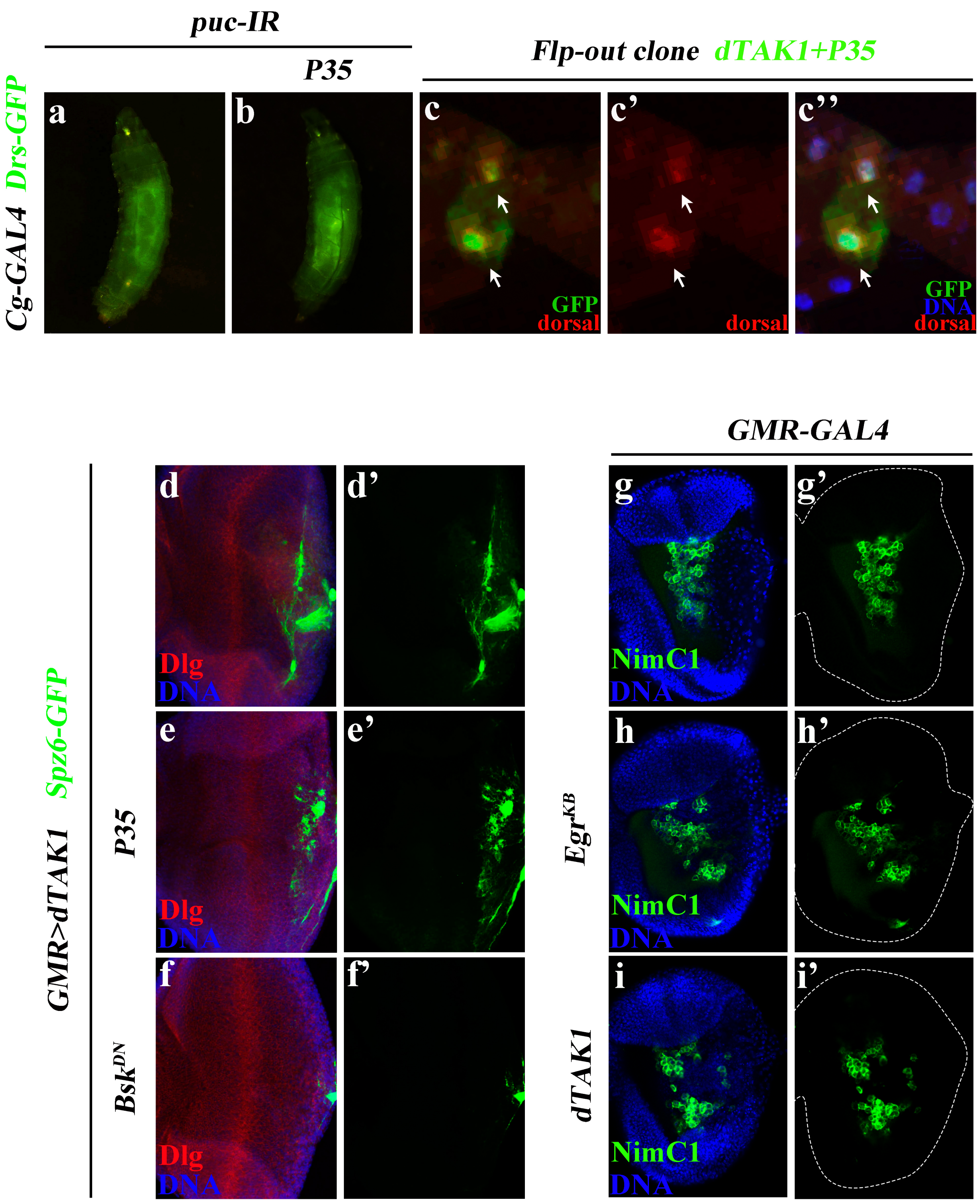

Wu et al., Figure S10

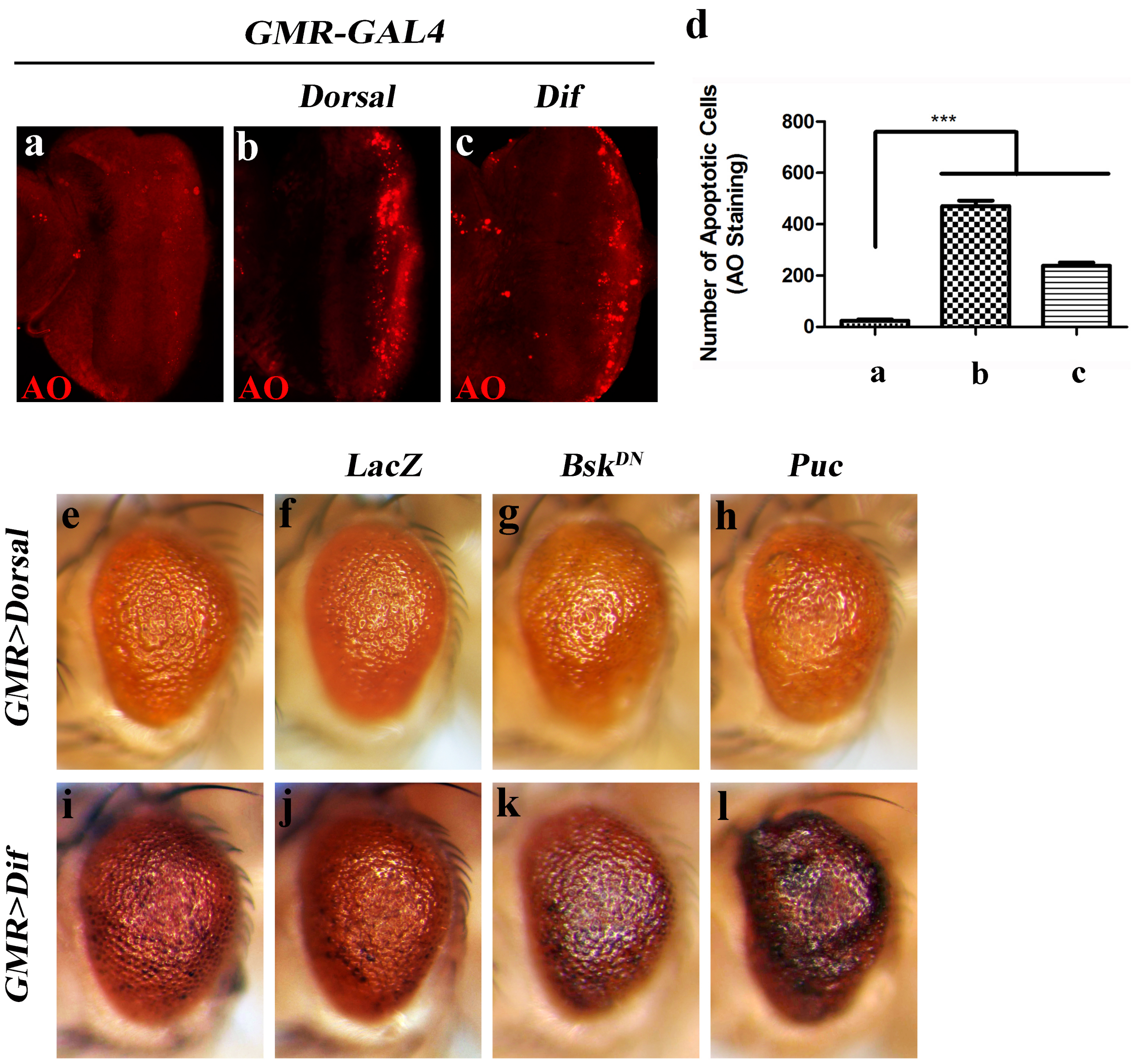

Wu et al., Figure S11

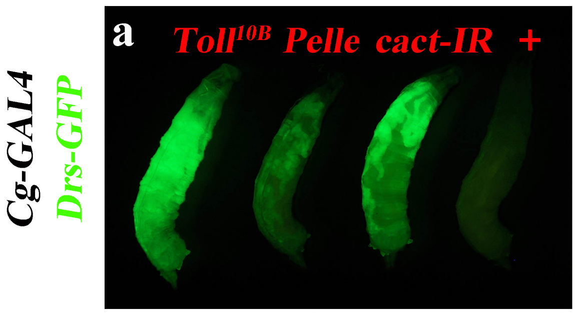

Wu et al., Figure S12

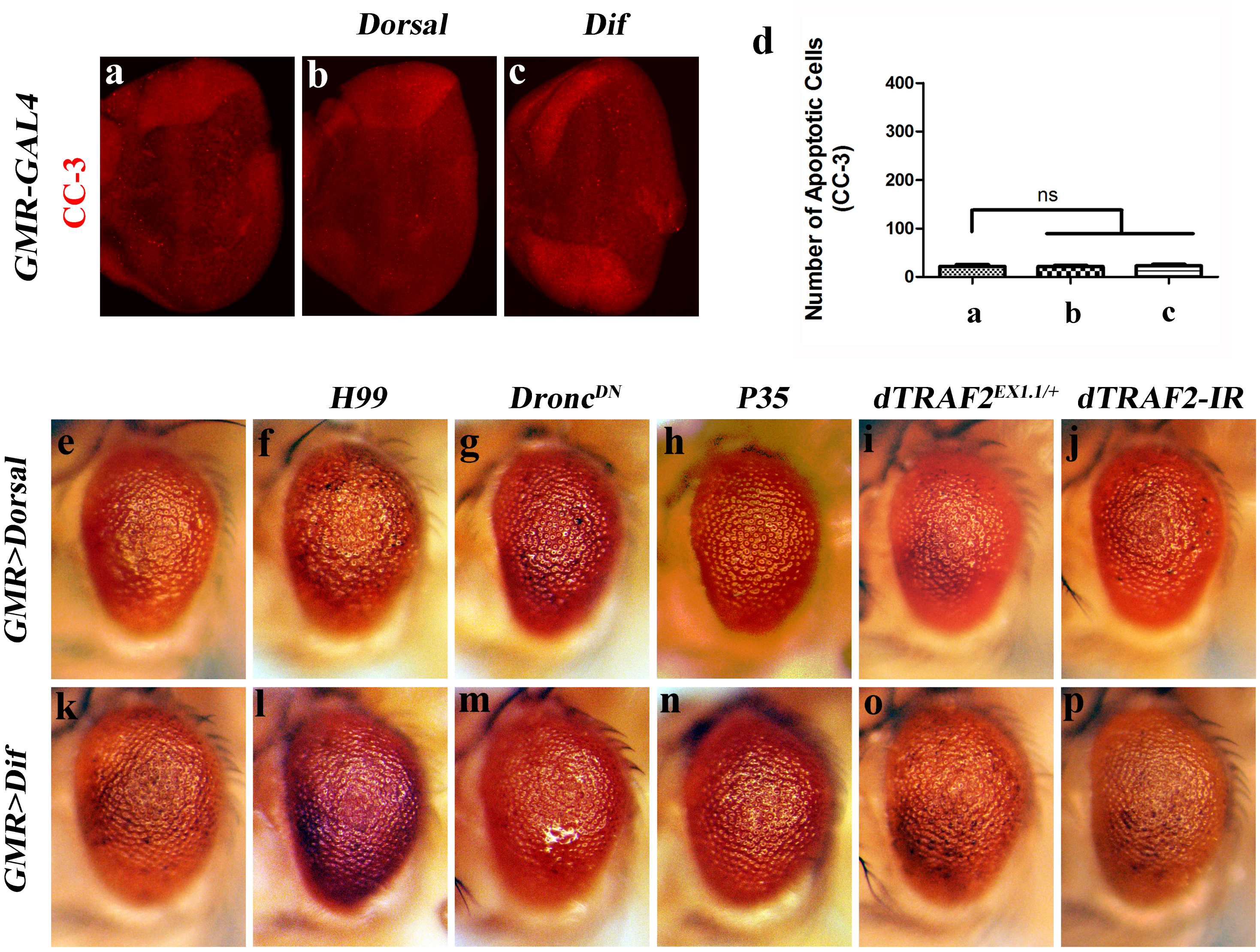

Supplement: Supplementary Information [file rsob140171supp1.pdf]
